# Ordinal Sparse Neural Networks for Modeling Gene- and Imaging-Environment Interactions

**DOI:** 10.1002/sim.70302

**Published:** 2025-10

**Authors:** Jiajing Xue, Yaqing Xu, Jingmao Li, Shuangge Ma, Kuangnan Fang

**Affiliations:** 1 Department of Statistics and Data Science, School of Economics, Xiamen University, Xiamen, Fujian, China; 2 School of Public Health, Shanghai Jiao Tong University School of Medicine, Shanghai, China; 3 Department of Biostatistics, Yale School of Public Health, New Haven, Connecticut, USA; 4 Fujian Key Laboratory of Statistical Science, Xiamen University, Xiamen, Fujian, China

**Keywords:** cancer modeling, high-dimensional data, interaction analysis, neural network, ordinal response

## Abstract

In biomedical studies, gene-environment (G-E) interactions and imaging-environment (I-E) interactions play an important role in modeling disease outcomes. Substantial investigations have been made; however, there is still a lack of related studies exploring flexible nonparametric statistical methods for modeling ordinal responses, such as the tumor pathological stage. In this paper, we develop a neural network-based method for modeling ordinal responses with interaction analysis. A novel definition of the output function for the neural network is derived to predict ordinal categories. To facilitate variable selection, we employ a sparse layer within the proposed neural networks. The penalized estimation is obtained using the local quadratic approximation (LQA) algorithm. Extensive simulation studies demonstrate that the proposed method achieves competitive performance in both prediction and variable selection. We further apply our method to breast cancer (BRCA) and skin cutaneous melanoma (SKCM) datasets, examining tumor stage prediction based on G-E and I-E interaction analyses, respectively. The proposed method identifies relevant main effects and interactions, providing insights into the underlying biological mechanisms.

## 1 | Introduction

In the past few decades, many cancer studies have demonstrated the important implications of interactions, including gene-environment (G-E) and imaging-environment (I-E) interactions [1, 2]. G-E interaction analysis has garnered extensive attention and led to numerous significant achievements. Recently, I-E interaction analysis has also gained traction, as histopathological imaging plays a critical role in disease diagnosis and can provide complementary insights to genomic and clinical data. In practical applications, researchers adopt different modeling strategies for interaction analysis. Some existing methods perform interaction analysis using a marginal model for each main effect and its corresponding interactions [3]. Others examine joint statistical modeling, in which the overall information and underlying biological mechanisms are taken into consideration. In the following analysis, we apply a joint modeling framework, as it can better reflect disease biology. Nevertheless, joint analysis may involve high-dimensional main effects and interactions, posing difficulties in modeling. Comprehensive and rigorous discussions are provided in previous studies [4, 5].

Numerous studies within the context of G-E and I-E interaction analyses investigate diverse disease outcomes, encompassing continuous, survival, and categorical responses [6–8]. Among these outcomes, ordinal response plays an important role in biomedical research, and examples include tumor pathological stage, blood pressure levels, depression severity, and Knodell score. A variety of parametric models exist for analyzing ordinal response data, including the cumulative logit model, adjacent-categorical logit model, and continuation ratio model, and are applied in both low-dimensional and high-dimensional settings. For instance, Lee examines applications to coronary arterial disease and postoperative wound infection based on the cumulative logit model [9]. Archer and Williams combine a penalization term and the continuation ratio model in modeling ordinal responses with gene expression data [10]. Baccianella et al. present four novel variable selection metrics for ordinal regression [11]. Gentry et al. propose a method for predicting the stage of breast cancer (BRCA) with high-dimensional covariates [12]. However, ordinal outcomes remain relatively underexplored, specifically under the nonlinear G-E/I-E interaction frameworks, despite their critical importance in biomedical research.

To facilitate more flexible modeling, many studies have developed nonparametric models for ordinal outcomes. For example, Hastie and Tibshirani develop a nonparametric additive proportional odds model, where the ordinal categories are intended to depict the age of the cell [13]. With a Bayesian perspective, DeYoreo and Kottas propose a nonparametric model for ordinal regression, avoiding assumptions of linearity or additivity in standard models [14]. Bao and Hanson model ordinal data as a finite stick-breaking mixture of multivariate probit models [15]. While these models substantially improve flexibility and generalizability, they typically do not incorporate structural constraints such as hierarchical principles, which are essential in high-dimensional interaction analysis [16]. In particular, a strong hierarchy, one of the common structures in biomedical research, implies that if an interaction term is identified, both corresponding main variables must also be identified in the model. Many statisticians advocate for preserving this constraint to ensure model interpretability and biological plausibility. Especially, a strong hierarchy is sensible when modeling G-E and I-E interactions, where main effects typically carry foundational biological meaning [16]. Therefore, there is a need for more expressive modeling tools that not only offer flexibility and nonlinearity but also provide a framework that can be extended to incorporate the strong hierarchical structure.

To make such an extension, we consider applying neural networks. Recently, neural networks and deep learning have emerged as powerful tools in various applications, due to their superior capability to approximate unknown functions. In biomedical research, they are successfully applied to a wide variety of tasks, including disease classification, prognosis prediction, and integrative multi-omics modeling [17, 18]. Despite their successes, applying neural networks to model ordinal outcomes with interactions remains challenging. First, existing neural networks are not designed to handle ordinal labels, as most output activation functions are tailored to regression or nominal classification tasks, ignoring the intrinsic ordering of categories. Second, few neural networks can incorporate structured second-order interaction terms, which are central to many biological and statistical models. Third, although substantial research has been conducted on variable selection within the neural network framework, covering both applications and theoretical developments [19, 20], these efforts primarily focus on main effects, with limited attention to enforcing hierarchical constraints between main and interaction effects.

In this paper, we introduce a sparse neural network for modeling ordinal responses with high-dimensional interactions. Specifically, our method contributes to the following areas. First, the proposed method addresses the problem of nonparametric ordinal modeling in the presence of high-dimensional interactions. Unlike existing nonparametric studies for ordinal response, we do not impose specific conditions on the form of the unknown function, such as additivity. Second, we derive a novel output function tailored to ordinal response modeling. The proposed output function enables the model to learn the unknown relationship between covariates and an ordinal response. Third, we adopt a sparse layer in the neural network to identify important main effects and interactions. A sparse group penalty is employed to enforce the strong hierarchical structure between main effects and interactions. Numerical results demonstrate the competitive performance of our method compared to alternatives. Moreover, we apply our method to two cancer types using data from The Cancer Genome Atlas (TCGA): G-E interactions for predicting BRCA stage and I-E interactions with histopathological imaging features for predicting SKCM stage.

The remainder of the paper is organized as follows. Section 2 introduces the proposed ordinal sparse neural network and its estimation procedure. Section 3 presents extensive simulation studies demonstrating the advantages of our method in both variable selection and prediction performance. In Section 4, we summarize the real data applications. In Section 5, we provide a discussion of our method and consider its potential development.

## 2 | Methods

In this section, we describe the proposed method in detail. Section 2.1 introduces the data and models, where we consider the nonparametric cumulative logit model for analyzing ordinal responses. The architecture of the proposed neural network is presented in Section 2.2. In Section 2.3, we derive the loss function using a sparse group penalty for model estimation and variable selection. The algorithm and computational details are summarized in Section 2.4.

### 2.1 | Problem Setting

Let ${𝒟}_{n}={\left\{\left(x_{i},z_{i},y_{i}\right)\right\}}_{i=1}^{n}$ denote the observed data, where $x_{i}=\left(x_{i1},\ldots ,x_{ip}\right)$ is a p-dimensional vector for G (or I) variables, and $z_{i}=\left(z_{i1},\ldots ,z_{iq}\right)$ is a q-dimensional vector for E variables. Response variable $y_{i}$ is ordinal with K ordered categories, labeled as 1,…,K. The nonparametric cumulative logit model is defined as follows:

$$y_{i}=k\;\text{if}\;a_{k-1}<u_{i}\leq a_{k},\;k=1,\ldots ,K,\;u_{i}=-f\left(x_{i},r_{i},z_{i}\right)+\epsilon_{i},$$

where $u_{i}$ is a latent continuous variable. Here, the interaction vector $r_{i}\in {\mathbb{R}}^{pq}$ is defined as $r_{i}=(x_{i1}z_{i1},\ldots ,x_{i1}z_{iq},\ldots ,x_{ip}z_{i1},\ldots ,x_{ip}z_{iq}{)}^{⊤},f\;:\;{\mathbb{R}}^{p+pq+q}\mapsto \mathbb{R}$ is an unknown nonlinear function, and $\epsilon_{i}$ denotes the error term. The threshold parameters $a_{k}$, for k=0,…,K, satisfy the ordering constraint $-\infty =a_{0}<a_{1}<\cdots <a_{K-1}<a_{K}=\infty$.

The cumulative logit model assumes that the ordinal nature of the observed response arises from methodological limitations in data collection. For example, Body Mass Index (BMI) is a positive, continuous variable that is often categorized into discrete intervals under certain clinical guidelines, resulting in an ordinal outcome [21]. Assuming that error term $\epsilon_{i}$ follows a logistic distribution, we obtain

(1) $$P\left(y_{i}\leq k|x_{i},z_{i}\right)=\frac{exp\left\{a_{k}+f\left(x_{i},r_{i},z_{i}\right)\right\}}{1+exp\left\{a_{k}+f\left(x_{i},r_{i},z_{i}\right)\right\}},\;k=1,\ldots ,K$$

Furthermore, the class-specific probabilities can be obtained by taking the differences between the consecutive cumulative probabilities:

$$p_{k}\left(x_{i},z_{i}\right)=P\left(y_{i}\leq k|x_{i},z_{i}\right)-P\left(y_{i}\leq k-1|x_{i},z_{i}\right),$$

where $p_{k}\left(x_{i},z_{i}\right)=P\left(y_{i}=k|x_{i},z_{i}\right)$ for k=1,…,K.

Our objectives are to accurately predict the ordinal categories based on the fitted model and to identify important main effects and interactions under the strong hierarchical constraint.

### 2.2 | Ordinal Sparse Neural Networks

**FIGURE 1 |:**
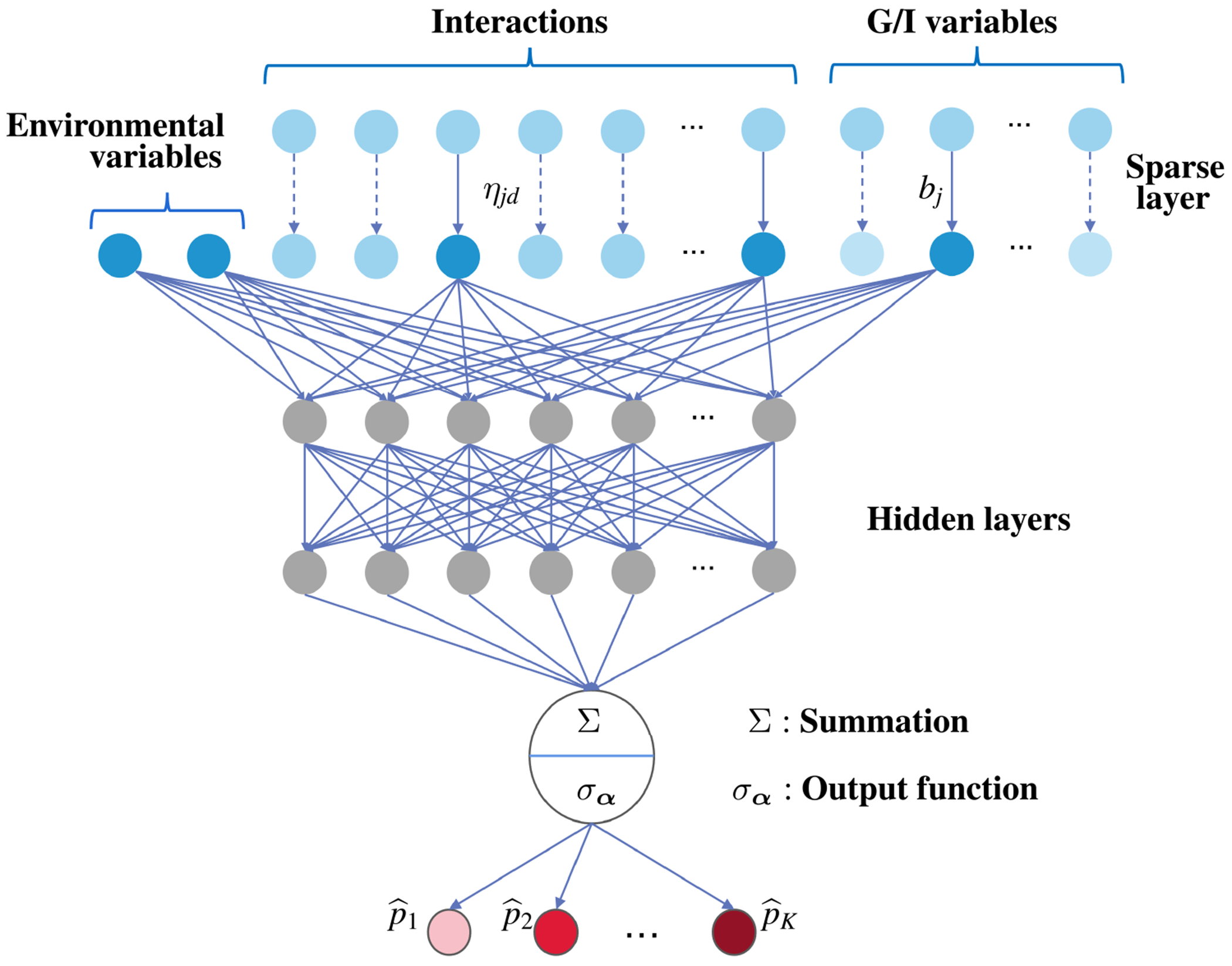
Architecture of the proposed ordinal sparse neural network, where ${\hat{p}}_{k}$ denotes the output probability for the *k*-th category, *k* = 1, …, *K*.

To estimate the nonparametric cumulative logit model, we employ a neural network due to its superior approximation capability [22]. The proposed ordinal sparse neural network consists of a sparse layer, L−1 hidden layers, an activation function ψ, a node-size vector $m=\left(m_{0},\ldots ,m_{L}\right)$, and a novel output function $\sigma_{\alpha }(\cdot )$. The network is defined as follows:

(2) $$N(t;\Theta ,\alpha )=\sigma_{\alpha }\left(W_{L}\psi \left(\cdots \psi \left(W_{1}(\beta \circ t)+\upsilon_{1}\right)\cdots \right)+\upsilon_{L}\right),$$

where input $t\in {\mathbb{R}}^{m_{0}}$ is an $m_{0}$-dimensional feature vector, ψ is the ReLU activation function, and ∘ denotes Hadamard (component-wise) product. Figure 1 illustrates the architecture of the proposed neural network. In this framework, we set $m_{0}=p+pq+q$ and $m_{L}=1$ to accommodate interactions. Parameter set Θ={β,θ} includes both the sparse layer coefficients and the neural network parameters. Here, θ denotes the collection of weights $W_{l}\in {\mathbb{R}}^{m_{l}\times m_{l-1}}$ and biases $v_{l}\in {\mathbb{R}}^{m_{l}}$ for l=1,…,L. Vector $\beta ={\left(b^{⊤},\eta^{⊤},1_{q}^{⊤}\right)}^{⊤}\in {\mathbb{R}}^{p+pq+q}$ represents the coefficients within the sparse layer. Specifically, $b={\left(b_{1},\ldots ,b_{p}\right)}^{⊤}$ and $\eta ={\left(\eta_{11},\cdots ,\eta_{pq}\right)}^{⊤}$ establish a one-to-one connection between each input variable and the subsequent network layers. Therefore, the G/I variables and interactions are weighted as ($b_{1}x_{1},\ldots ,b_{p}x_{p}$) and $\left(\eta_{11}x_{1}z_{1},\cdots ,\eta_{pq}x_{p}z_{q}\right)$, respectively. The all-ones vector $1_{q}\in {\mathbb{R}}^{q}$ assigns unit weights to the environmental variables, which are assumed to be pre-selected and low-dimensional. The definition of vector β plays a crucial role in variable selection. Variables with corresponding coefficients equal to zero are considered unimportant and excluded from subsequent computations. To accommodate ordinal response, we define a novel output function $\sigma_{\alpha }\;:\;\mathbb{R}\mapsto (0,1{)}^{K}$. Specifically, the k-th output of $\sigma_{\alpha }$ is defined as follows:

$${\left[\sigma_{\alpha }(x)\right]}_{k}=\frac{\text{exp}\left(\alpha_{k}+x\right)}{1+\text{exp}\left(\alpha_{k}+x\right)}-\frac{\text{exp}\left(\alpha_{k-1}+x\right)}{1+\text{exp}\left(\alpha_{k-1}+x\right)},\;k=1,\ldots ,K,$$

where the notation $[\cdot {]}_{k}$ denotes the k-th component of the vector. Parameter vector $\alpha =\left(\alpha_{1},\ldots ,\alpha_{K-1}\right)$ satisfies the monotonicity constraint $-\infty =\alpha_{0}<\alpha_{1}<\cdots <\alpha_{K}=\infty$. Moreover, the definition guarantees that $\sum_{k=1}^{K}{\left[\sigma_{\alpha }(x)\right]}_{k}=1$. Let ${\hat{p}}_{k}\left(x_{i},z_{i}\right)$ denote the kth output of (2), where the input vector is $t=\left(x_{i},r_{i},z_{i}\right)$. In the following, we use ${\hat{p}}_{k}\left(x_{i},z_{i}\right)$ to model the probability of the kth category.

### 2.3 | Penalized Estimation

To estimate the proposed ordinal sparse neural network and conduct variable selection under the strong hierarchical constraint, we propose the following loss function:

(3) $$𝒬\left(\phi ;{𝒟}_{n}\right)=ℒ\left(\phi ;{𝒟}_{n}\right)+𝒫(\text{Θ};\lambda ,\lambda_{1},\lambda_{2},\xi )$$

$$ℒ\left(\phi ;{𝒟}_{n}\right)=-\frac{1}{n}\sum_{i=1}^{n}\sum_{k=1}^{K}{\overset{˘}{y}}_{ik}\;\text{log}\;{\hat{p}}_{k}\left(x_{i},z_{i}\right)$$

(4) $$𝒫(\text{Θ};\lambda ,\lambda_{1},\lambda_{2},\xi )={\text{pen}}_{1}+{\text{pen}}_{2},\;{\text{pen}}_{1}=\sum_{j=1}^{p}\rho \left(\|\kappa_{j}{\|}_{2};\lambda_{1},\xi \right)+\sum_{j=1}^{p}\sum_{d=1}^{q}\rho \left(\eta_{jd};\lambda_{2},\xi \right)\;{\text{pen}}_{2}=\lambda \sum_{l=1}^{L}\|W_{l}{\|}_{F}^{2}$$

where ϕ={α,Θ} is the collection of parameters to be optimized, $\kappa_{j}={\left(b_{j},\eta_{j1},\cdots ,\eta_{jq}\right)}^{⊤}$ is the (q+1)-dimensional vector corresponding to the jth main factor and its interactions, $\|\cdot {\|}_{2}$ represents the $\ell_{2}$ norm of a vector, and $\|\cdot {\|}_{F}$ denotes the Frobenius norm of a matrix. Here, ${\overset{˘}{y}}_{ik}=1\left(y_{i}=k\right)$ is the kth element of ${\overset{˘}{y}}_{i}$, which denotes the one-hot encoding vector for $y_{i}$.

In the loss function (3), the first term $ℒ\left(\phi ;{𝒟}_{n}\right)$ is the crossentropy loss that quantifies the discrepancy between the true label distribution and the predicted probabilities, and the second term is the penalty. In Equation (4), the term ${\mathrm{pen}}_{1}$ denotes the sparse group penalty. Here, function $\rho (t;\lambda ,\xi )=\lambda \int_{0}^{\left|t\right|}(1-x/\lambda \xi {)}_{+}dx$ is the minimax concave penalty (MCP) [23]. This penalty term is designed to enforce the hierarchical structure between main effects and interactions. Specifically, if the *j*th group is not selected, all the associated interaction terms are treated as irrelevant. Conversely, if the *j*th group is selected, the penalty proceeds to assess each interaction coefficient to determine its importance. Under this definition, our neural network-based method achieves variable selection for main effects and interactions while maintaining the hierarchical structure. The term pen_2_ corresponds to ridge regularization, which is employed to prevent overfitting [24].

### 2.4 | Computation

A key challenge in optimizing the loss function (3) is maintaining the ascending order of parameter ***α***. Without appropriate constraints, this ordering may not be preserved. To address this, we adopt a reparameterization strategy that ensures the ordinal constraint is implicitly satisfied.

Specifically, we reparameterize α via an unconstrained vector $\gamma =\left(\gamma_{1},\ldots ,\gamma_{K-1}\right)$ as follows:

$$\alpha_{1}=\gamma_{1},\;\alpha_{k}=\alpha_{1}+\sum_{j=2}^{k}exp\left(\gamma_{j}\right),\;\text{for}\;k=2,\cdots ,K-1,$$

where each parameter $\gamma_{j}\in \mathbb{R}$ can be initialized from normal distribution 𝒩(0, 4). This reparameterization allows γ to vary freely without constraints, while the order of $\alpha_{k}$ is naturally maintained throughout the training process.

Having established the reparameterization to handle the ordinal constraint, we now optimize the loss function (3) using the local quadratic approximation (LQA) algorithm [25]. In what follows, superscript [m] denotes the mth iteration. We define ${\tilde{pen}}_{1}$ as the quadratic approximation of the penalty term ${\mathrm{pen}}_{1}$, given by

$${\tilde{\text{pen}}}_{1}=\sum_{j=1}^{p}\frac{\rho_{\lambda_{1},\xi }^{\prime }\left(\|\kappa_{j}^{\left[m\right]}{\|}_{2}\right)}{2\|\kappa_{j}^{\left[m\right]}{\|}_{2}}\frac{\left|b_{j}^{\left[m\right]}\right|}{\|\kappa_{j}^{\left[m\right]}{\|}_{2}}b_{j}^{2}\;+\sum_{j=1}^{p}\sum_{d=1}^{q}\left(\frac{\rho_{\lambda_{1},\xi }^{\prime }\left(\|\kappa_{j}^{\left[m\right]}{\|}_{2}\right)}{2\|\kappa_{j}^{\left[m\right]}{\|}_{2}}\frac{\left|\eta_{jd}^{\left[m\right]}\right|}{\|\kappa_{j}^{\left[m\right]}{\|}_{2}}+\frac{\rho_{\lambda_{2},\xi }^{\prime }\left(\eta_{jd}^{\left[m\right]}\right)}{2\left|\eta_{jd}^{\left[m\right]}\right|}\right)\eta_{jd}^{2},$$

where $\rho_{\lambda ,\xi }^{\prime }(t)=sign(t)\cdot (\lambda -|t|/\xi {)}_{+}$ is the first order derivative of the MCP penalty. Within our framework, we reformulate function $\tilde{𝒬}\left(\phi ;{𝒟}_{n}\right)=ℒ\left(\phi ;{𝒟}_{n}\right)+{\tilde{pen}}_{1}+{pen}_{2}$ and minimize it iteratively.

Given the parameters and reparameterized $\gamma^{\left[m\right]}=(\gamma_{1}^{\left[m\right]},\cdots ,\gamma_{K-1}^{\left[m\right]})$ at the mth iteration, we update α iteratively using the following steps:

(5) $$\gamma^{\left[m+1\right]}=\gamma^{\left[m\right]}-\zeta \frac{\partial \tilde{𝒬}\left(\phi^{\left[m\right]};{𝒟}_{n}\right)}{\partial \gamma^{\left[m\right]}}$$

(6) $$\{\begin{array}{l} \alpha_{1}^{\left[m+1\right]}=\gamma_{1}^{\left[m\right]},\\ \alpha_{k}^{\left[m+1\right]}=\alpha_{1}^{\left[m\right]}+\sum_{j=2}^{k}\text{exp}\left(\gamma_{j}^{\left[m\right]}\right),\;k=2,\ldots ,K-1, \end{array}$$

where ζ represents the learning rate. The update steps of $\Theta^{\left[m\right]}=\left\{\beta^{\left[m\right]},\theta^{\left[m\right]}\right\}$ and complete iterative optimization procedure are summarized in Algorithm 1.

**Remark.** We highlight several practical considerations. First, the initialization of Θ^[0]^ with nonzero values is essential, as commonly practiced in neural network literature. Second, the estimated coefficients may not attain exact zeros due to the use of smooth approximations, as noted in related studies [26]. To address this, a small threshold is applied to truncate negligible estimates, which does not violate the strong hierarchical structure. Third, although additional techniques such as dropout can be employed to mitigate overfitting, we adopt ridge regularization on the weight matrices in our numerical study.

With the optimization algorithm in place, we now discuss several implementation details, including the neural network architecture and selection of tuning parameters. In our practice, we

**ALGORITHM 1 |:** Optimization based on LQA.

**Require:** Observations ${𝒟}_{n}$, learning rate ζ, tuning parameters $\lambda ,\lambda_{1},\lambda_{2},\xi$.
Initialize $\phi^{\left[0\right]}$ and *m* = 0,
**repeat**
compute ***α***^[*m*+1]^ by (5–6),
compute $\theta^{\left[m+1\right]}=\theta^{\left[m\right]}-\zeta \frac{\partial \tilde{Q}(\phi^{\left[m\right]};{𝒟}_{n})}{\partial \theta^{\left[m\right]}}$,
**for** *j* = 1, …, *p* **do**
$b_{j}^{\left[m+1\right]}=b_{j}^{m}-\zeta \frac{\partial \tilde{Q}(\phi^{\left[m\right]};{𝒟}_{n}}{\partial b_{j}^{\left[m\right]}}$,
**for** *d* = 1, …, *q* **do**
$\eta_{jd}^{\left[m+1\right]}=\eta_{jd}^{\left[m\right]}-\zeta \frac{\partial \tilde{𝒬}\left(\phi^{\left[m\right]};{𝒟}_{n}\right)}{\partial \eta_{jd}^{\left[m\right]}}$,
**end for**
**end for**
m←m+1,
**until** convergence or *m* reaches its maximum *M*.
**Ensure:** Estimates for ϕ.

adopt a sparse ordinal neural network with two hidden layers and 128 nodes per layer, in line with previous studies on high-dimensional neural networks [27]. Hyperparameter *ξ* is fixed at 3, as recommended in Zhang [23], and tuning parameters *λ*, *λ*_1_, and *λ*_2_ are selected based on prediction performance on a held-out validation set. A grid search is conducted for all tuning parameters.

## 3 | Simulation Study

We conduct a simulation to evaluate the performance of the ordinal sparse neural network. We generate G (or I) variables from a 100-dimensional multivariate normal distribution ${𝒩}_{100}(0,\Sigma (\rho ))$, where matrix Σ(ρ) follows an autoregressive (AR) structure, such that the correlation between the sth and tth variables is $\rho^{\left|s-t\right|}$. We generate five correlated normal variables from ${𝒩}_{5}(0,\Sigma (0.5))$ and then dichotomize two of these variables at 0, resulting in five environmental variables, three continuous and two discrete. The dimensionality of the important variables is 15 for both main effects and interactions, in accordance with the strong hierarchical structure. In the following simulations, we examine nonlinear and linear examples under varying proportions of categories, with n=300, 500, 700 and ρ=0.0, 0.25.

**Example 1** (Nonlinear setting). This example considers a nonlinear setting. The latent variable *u_i_* is defined as:

$$u_{i}=\sum_{j=1}^{15}\omega_{j}x_{ij}+\sum_{j=1}^{15}c_{j1}x_{ij}z_{i1}+\sum_{d=1}^{5}s_{d}z_{id}\;+\text{sin}\left(\sum_{j=1}^{15}\omega_{j}x_{ij}+\sum_{j=1}^{15}c_{j1}x_{ij}z_{i1}+\sum_{d=1}^{5}s_{d}z_{id}\right)+\epsilon_{i},$$

where coefficients $\omega_{j},c_{j1}$ and $s_{d}$ are independently drawn from Uniform distribution 𝒱(0.6, 0.9). Error term $\epsilon_{i}$ follows the standard Logistic distribution ℒ(0, 1), as defined in Equation (A1) in Appendix A. Ordinal response $y_{i}$ is generated by discretizing $u_{i}$ based on thresholds $a_{0},\ldots ,a_{3}$. Here, $a_{0}=-\infty$ and $a_{3}=\infty$, and the category proportions are controlled by adjusting $a_{1}$ and $a_{2}$.

For instance, setting $a_{1}$ and $a_{2}$ to the 1/3 and 2/3 quantiles of $u_{i}$ results in approximately balanced category proportions, that is, 1:1:1. To evaluate performance under weaker interaction signals, we additionally consider a setting where $c_{j1}∼𝒰(0.4, 0.7)$ with ρ=0. In subsequent analyses, we vary the values of $a_{1}$ and $a_{2}$ to generate imbalanced categories. Additional settings, including Examples B1 and B2, are provided in Appendix B.

To facilitate comparison, we consider the following alternative methods.

- Alt.1: It considers a similar sparse neural network and penalized estimation as the proposed method, but uses Softmax as the output function. Therefore, the category labels are treated as nominal rather than ordinal. Comparing with this approach can establish the merit of the proposed output function *σ*_***α***_.
- Alt.2: This approach uses the same sparse neural network and output function *σ*_***α***_ as the proposed method, but applies a penalty to each coefficient, without enforcing the strong hierarchical structure. Comparing with this approach can demonstrate the importance of incorporating the hierarchical structure.
- Alt.3: It uses the same architecture and output function *σ*_***α***_, but excludes the sparse layer. Thus, there is no penalization term in the loss function, and all covariates are utilized in the model. This analysis can help assess the necessity of the sparse layer and variable selection.
- Alt.4: The sparse group penalized ordinal regression is conducted. This method may serve as a parametric baseline.
- Alt.5: Marginal analysis is employed, in which one G/I variable, all E variables, and their interactions are examined at a time. *p* values are used for variable selection. This analysis serves as a benchmark, given the popularity of marginal analysis.
- Alt.6: The ordered random forest method is applied [28]. This nonparametric approach is tailored for ordinal responses. This alternative allows us to compare with an established nonlinear baseline.

For the alternative methods, the tuning parameters are also selected using a held-out validation set and grid search, following a process similar to that of the proposed approach. While other applicable methods exist, the six discussed above are more directly relevant to this study.

We evaluate performance in variable selection and prediction. For variable selection, methods are compared using true positive rate (TPR) and false positive rate (FPR). For prediction performance, we use the following evaluation metrics. To account for class imbalance, we use macro-AUC, which computes the unweighted average of AUC scores from one-vs-rest comparisons across classes, ensuring that minority classes are treated equally in evaluation [29]. To reflect the ordinal nature of categories, we calculate the Ranked Probability Score (RPS), which quantifies the differences between the predicted cumulative class probabilities and the observed cumulative distribution of outcomes [30]. In addition, we use accuracy, a commonly adopted metric for binary and multi-classification problems. Higher macro-AUC, higher accuracy, and lower RPS indicate better prediction performance. The definitions of macro-AUC and RPS are summarized in Appendix A.

**FIGURE 2 |:**
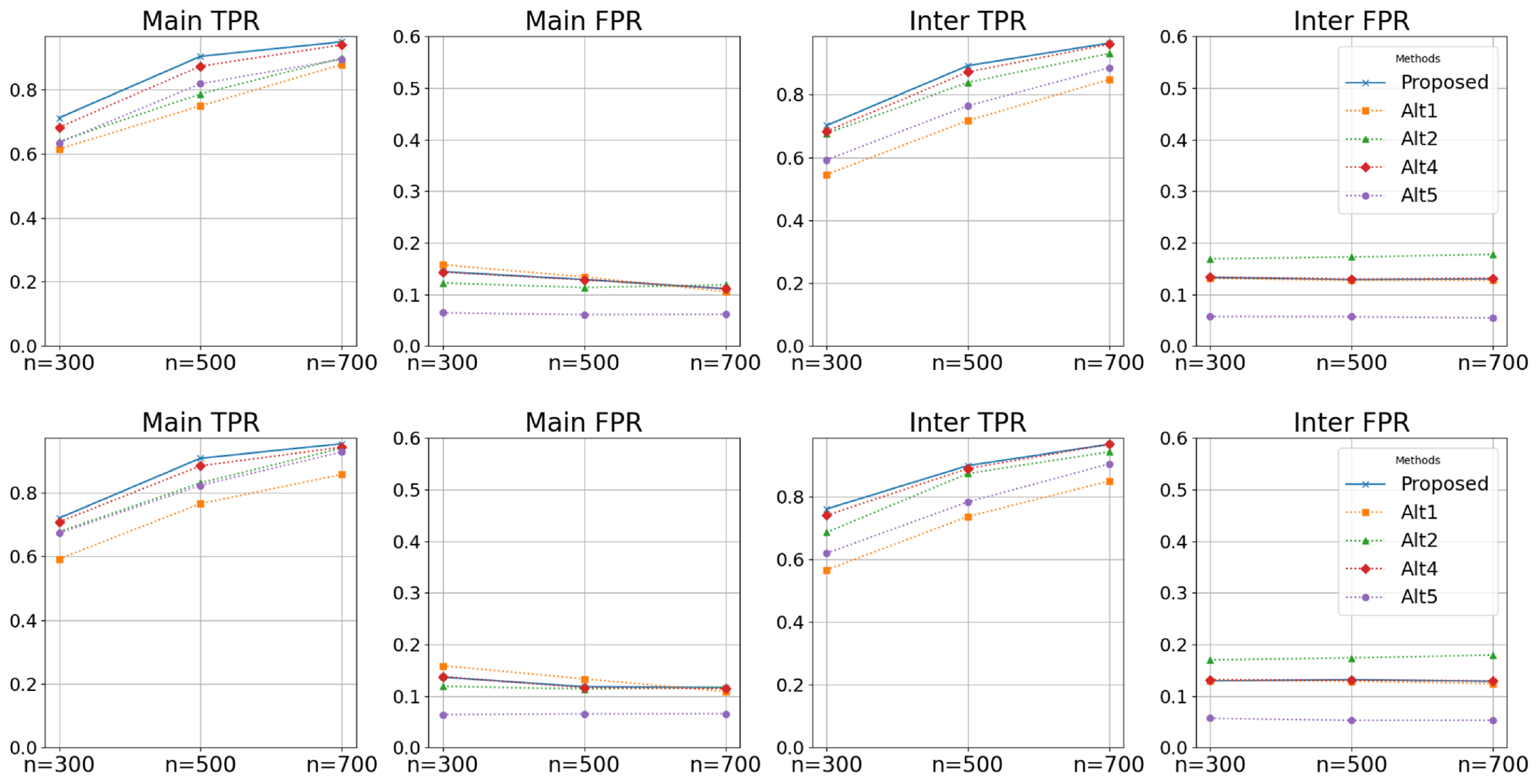
Simulation results for variable selection in Example 1 with AR(0.0). The top and bottom rows correspond to response class proportions of 1 : 1 : 1 and 1 : 2 : 1, respectively. “Main” refers to main effects, and “Inter” to interactions.

We present simulation results based on 100 replicates in Table 1, Figure 2, and Supporting Information. Overall, the proposed approach demonstrates competitive performance in both variable selection and prediction across all the simulation settings. Specifically, the performance of all methods improves as sample size increases, with our method consistently outperforming the others. For prediction, the proposed method achieves higher macro-AUC, higher accuracy, and lower RPS. For instance, in Table 1, under Example 1 with *ρ* = 0.25, *n* = 300, and balanced proportions, the proposed method has (macro-AUC, RPS, accuracy) = (0.752, 0.359, 0.553), compared with (0.694, 0.378, 0.517) for Alt.1, (0.727, 0.369, 0.530) for Alt.2, (0.727, 0.385, 0.533) for Alt.3, (0.719, 0.424, 0.533) for Alt.4, and (0.708, 0.376, 0.536) for Alt.6. Note that Alt.5 does not yield a prediction model. For variable selection, our approach can identify more true main effects and interactions while having a smaller or comparable FPR. For example, Figure 2, under Example 1 with *ρ* = 0.0, demonstrates the trends of the proposed method under increasing sample size, shown by solid lines. The better performance of our approach over Alt.1 underscores the necessity of the proposed output function. The comparison between the proposed approach and Alt.2 confirms the necessity of the hierarchical structure. Alt.3 and Alt.6 include both signals and noises, which can lead to inferior prediction performance, demonstrating the need for penalization. Furthermore, when comparing the proposed method to Alt.4, a linear model, it becomes evident that our method excels at capturing unknown and nonlinear relationships. Additional results are presented in the Supporting Information. Despite minor numerical differences, the overall patterns remain consistent with those discussed above.

## 4 | Real Data Applications

### 4.1 | G-E Interaction Analysis

Breast cancer is the most prevalent malignancy affecting women globally and poses a significant threat to their health and lives [31]. Between 2010 and 2019, the incidence of breast cancer increased at an average annual rate of approximately 0.5%. Recent studies indicate that breast cancer ranks as the leading cancer type among women, accounting for 31% of all cancer diagnoses in the female population. We conduct a G-E interaction analysis using the Cancer Genome Atlas (TCGA) breast cancer dataset. TCGA, curated by the National Institutes of Health (NIH), is a high-quality, publicly accessible dataset that spans multiple cancer types. Its comprehensiveness in genomic, transcriptomic, and proteomic data has made it an invaluable resource for cancer research.

**FIGURE 3 |:**
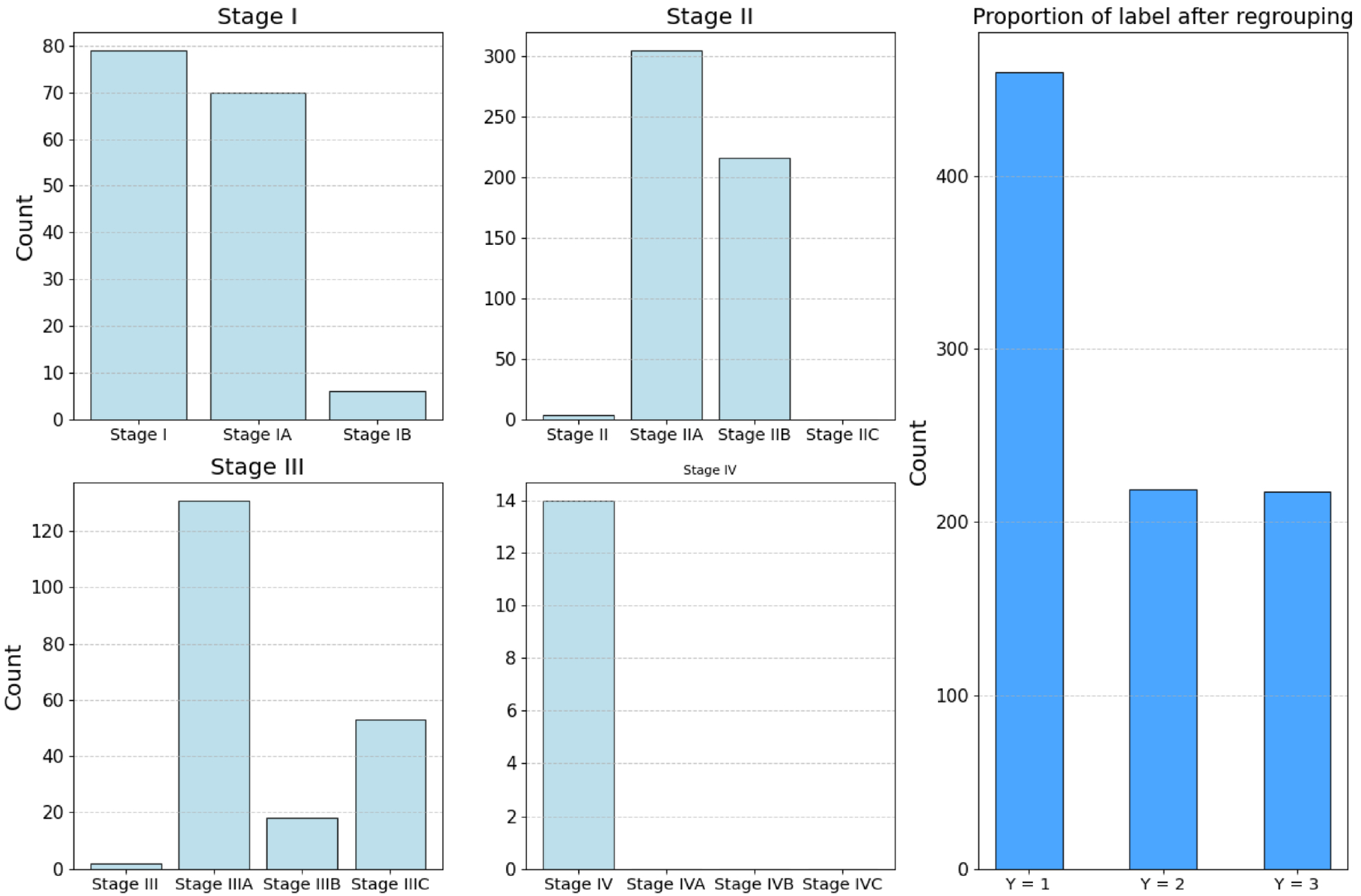
Original counts and regrouped labels for the BRCA data. The four panels on the left show the number of patients diagnosed at different cancer stages, while the panel on the right shows the proportions after regrouping.

The response variable is cancer stage, defined as the Neoplasm Disease Stage according to the American Joint Committee on Cancer Code. This is an ordinal variable that records cancer progression and indicates tumor severity. The distribution of the response variable is summarized in the four left panels of Figure 3. Given the imbalanced sample sizes across stages, we follow Wang et al. [32] and combine several adjacent stages to create three consolidated groups. The counts of the new labels after regrouping are shown in the right panel of Figure 3. Specifically, the rule of regrouping is as follows: Stages I, IA, IB, II, and IIA are combined into the first group, labeled as *Y* = 1; Stage IIB forms the second group, labeled as *Y* = 2; and Stages III to IV are combined into the third group, labeled as *Y* = 3.

For G variables, we use mRNA gene expression data obtained using the Illumina HiSeq RNA-Seq V2 platform. For E variables, we include age at diagnosis, estrogen receptor (ER) status assessed by immunohistochemistry (IHC), progesterone receptor (PR) status assessed by IHC, and human epidermal growth factor receptor 2 (HER2) status. These factors are widely recognized as significant for breast cancer [33]. Among the four pre-selected environmental variables, age at diagnosis is continuous, while ER, PR, and HER2 status are discrete variables. After matching the response variable with G and E variables and completing the necessary processing steps, the dataset for analysis includes 894 observations, 20 531 G variables, and 4 E variables. Given the ultra-high dimensionality of the G variables relative to the sample size, we employ a supervised screening method based on linear cumulative regression to improve model performance and computational efficiency. By extracting *p* values from the screening procedure and controlling FDR at 0.07, the number of G variables is reduced to 235.

We report 28 important genes and 27 interactions identified by the proposed model in Table 2. Through literature review, we find that the majority of the identified genes and interactions are supported by existing studies as related to BRCA. For instance, a member of the renin-angiotensin system (RAS), Angiotensin-converting enzyme 2 (ACE2), is reported to inhibit breast cancer angiogenesis. Moreover, ACE2 expression is negatively correlated with metastasis from breast cancer. ACSS2 is a nucleocytosolic enzyme responsible for converting acetate into acetyl-CoA. Its expression is upregulated in response to hypoxic conditions and nutrient deprivation stress. ABI1 dysregulation has been implicated in several cancers. In breast cancer, its expression is notably elevated, highlighting tissue- and disease-specific pathways through which ABI1 contributes to oncogenic transformation. This underscores the need for detailed mechanistic studies to better understand its role in cancer progression. Low expression of ACACB has been reported in laryngeal squamous cell carcinoma, triple-negative breast cancer, retinoblastoma, thyroid cancer, and melanoma. ACO2, which is significantly correlated with ACADS, can suppress proliferation and reduce the Warburg-like bioenergetic features of tumor cells in breast cancer. Additionally, adiponectin has been shown to decrease breast cancer cell proliferation in vitro while also lowering ACADS expression levels. This indicates that ACADS can serve as a potential biomarker for breast cancer. Activated liver X receptor (LXR) and retinoid X receptor (RXR) heterodimers drive the expression of genes that regulate cholesterol metabolism, such as the ATP-binding cassette (ABC) transporter ABCG1, which aids in the transfer of cholesterol from cells to high-density lipoprotein (HDL). Some studies suggest that LXR activation in MCF-7 breast cancer cells may deplete cholesterol by promoting its efflux, leading to the inhibition of cell proliferation and the induction of apoptosis. Beyond the genes discussed above, the identified genes also contain potential novel candidates such as ASIC5. Acid-sensing ion channels (ASICs) are a key class of acid sensors in cells, with ASIC1 playing a role in breast cancer pathogenesis by responding to the acidic tumor microenvironment. While ASIC5 is primarily expressed in the small intestine, its function remains unclear. Identifying ASIC5 can offer valuable insights into the underlying mechanisms. There are also several supportive studies for the identified interactions. For example, the expression of ABI1 is reported to show a significant positive correlation with age at diagnosis, which may explain the interaction effect between the two in relation to breast cancer progression. Additionally, some studies have investigated the relationship between ATP-binding cassette (ABC) transporter genes, such as ABCA and ABCB, and triple-negative breast cancer. This suggests that the interaction effects between the expressions of these genes and environmental variables like ER, PR, and HER2 should be given attention. As shown in Table 2, the interactions of ABCG1 with ER status, PR status, and those of ABCA6 with PR status and HER2, among others, are also highlighted.

To compare our method with the alternatives, we randomly split the data into training, validation, and testing sets with a ratio of 3:1:1. The validation set is used for selecting the tuning parameters, and the testing set is used for evaluating each method. The mean values (and standard deviations) of prediction based on 100 random splits are summarized in Table C1 in Appendix C. The proposed approach has (macro-AUC, RPS, accuracy) = (0.622, 0.413, 0.535), compared with (0.584, 0.434, 0.472) for Alt.1, (0.615, 0.424, 0.529) for Alt.2, (0.604, 0.428, 0.491) for Alt.3, (0.591, 0.431, 0.487) for Alt.4, and (0.603, 0.412, 0.498) for Alt.6. The improved prediction performance can provide some support to the proposed analysis. The overlapping identifications among the methods and the RV coefficients are summarized in Table 3. RV coefficient can measure overlapping information between two matrices, ranging from 0–1, with higher values indicating more overlap [34]. To evaluate identification, we adopt the observed occurrence index (OOI), which reflects the stability of identification across the splittings. The OOI of each gene is calculated by dividing the number of identifications by the number of splittings. A higher OOI indicates that a gene is identified more frequently across the splittings. We provide selected OOI values in Table C2. Most of the identified variables exhibit high OOI values, suggesting stability. For example, gene ACE2 and its interaction with diagnosis age are identified in all random splittings (OOI = 1.00), demonstrating the stability of ACE2 and this interaction.

### 4.2 | I-E Interaction Analysis

Histopathological imaging plays a critical role in cancer diagnosis and research. Radiologists routinely use various imaging modalities, such as Magnetic Resonance Imaging (MRI), Computed Tomography (CT), and others, to obtain detailed images of tumors and surrounding tissues. These imaging techniques offer a non-invasive means to evaluate tumor characteristics, monitor disease progression, and assist in treatment planning. Histopathological images, in particular, allow examination of tissue at a microscopic level, revealing key features such as tissue architecture, which is essential for accurate diagnosis. Widely regarded as the gold standard for cancer diagnosis, histopathological images reveal detailed micro-level characteristics of tumors. Some studies have modeled cancer outcomes using imaging features [35].

We conduct an I-E interaction analysis for SKCM, which is one of the most common forms of skin cancer and presents a significant challenge for early detection and personalized treatment strategies. Imaging features (I variables) are derived from the diagnostic slides obtained from the TCGA portal. The original whole slide histopathological images are too large for direct analysis. Therefore, we crop the images and save them as TIFF files using the OpenSlide Python library. This cropping procedure is common in image processing workflows and has been widely adopted in the literature. Furthermore, we use the software Cell-Profiler, a platform designed for cell image analysis, to extract quantitative features from each sub-image. After measuring cell-level features, we obtained 226 imaging features (I variables), including area fraction, neighboring structure, and other relevant measurements. For each patient, the imaging features are normalized based on the sample mean at the patient level. Missing values with a missing rate of less than 20% are imputed using sample medians. The details for extracting imaging features can be found in Xu et al. [2].

As for E variables, we include age at diagnosis, gender, aneuploidy score (AS), fraction genome altered (FG), and mutation count (MC). While the overall incidence rate of SKCM has remained stable over the years, notable differences persist across various age and gender groups. For instance, younger individuals tend to exhibit a lower incidence compared to older populations, and males generally show a higher incidence than females. Moreover, in cancer research, aneuploidy is one of the characteristics of many cancers, indicating that during cell proliferation, chromosomal losses or gains may occur, leading to genomic instability. This instability in chromosome number is closely related to tumor development and malignant transformation. Additionally, FG is an important metric used to measure genomic alterations in tumors. It reflects the genomic instability of a tumor by quantifying the proportion of the genome that has undergone variations. A high FG value is typically associated with greater malignancy, invasiveness, and poorer prognosis, and may serve as a reference for tumor prognosis and personalized treatment. MC refers to the number of mutations detected in the genome, typically used to reflect the genomic instability of tumor cells. It serves as a prognostic marker in cancer research, helping assess the malignancy of the tumor, response to imunotherapy, and potential for targeted therapies. Several studies have shown a correlation between these indicators and SKCM.

**FIGURE C1 |:**
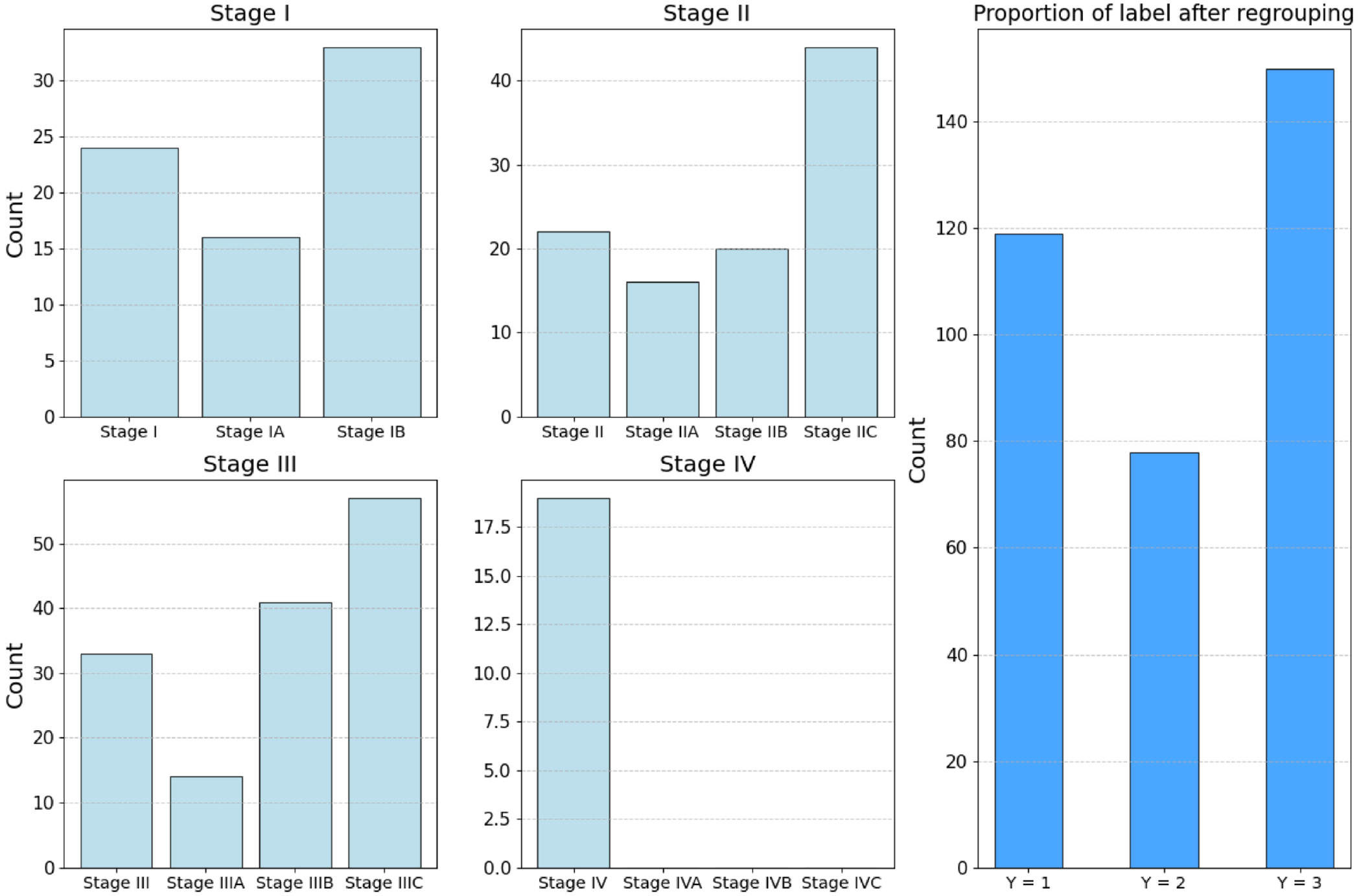
Original counts and regrouped labels for the SKCM data. The four panels on the left show the number of patients diagnosed at different cancer stages, while the panel on the right shows the proportions after regrouping.

The ordinal response is cancer stage, whose original and regrouped proportions are summarized in Figure C1. We regroup stage I, IA, IB, stage II, IIA, and stage I/II into the first group and relabel as *Y* = 1. Stage IIB, IIC, and IIIA make up the second group, which is labeled as *Y* = 2, and the rest are labeled as *Y* = 3. After the necessary processing steps, the total dimensionality of the variables is 1361 (comprising 226 imaging features, 1130 interactions, and 5 environmental variables), with a total of 347 observations.

Modeling the SKCM dataset results in the identification of 25 main imaging features and 34 interactions. The detailed identifications are provided in Supporting Information. Notably, the selected imaging features span multiple categories, including the “Geometry” group (e.g., AreaShape_Zernike_8_6, AreaShape_Orientation), which contains features that describe geometric properties (such as area and perimeter), as well as those derived from Zernike moments. Moreover, the “Texture” group (e.g., Texture_Correlation_ImageAfterMath_3_02_256) contains Haralick, Gabor wavelet, and Granularity features that characterize the textural properties of cells and tissues. As the detailed biological implications of high-dimensional imaging features remain less well understood compared to clinical and molecular data [36]. To facilitate further comparison, we perform 100 random splittings, and the average values of (macro-AUC, RPS, accuracy) are (0.553, 0.472, 0.426) for the proposed approach, (0.428, 0.483, 0.416) for Alt.1, (0.421, 0.481, 0.410) for Alt.2, (0.427, 0.485, 0.414) for Alt.3, (0.414, 0.480, 0.414) for Alt.4, and (0.582, 0.478, 0.423) for Alt.6. The improvement in prediction offers supporting evidence for the proposed analysis. The results are summarized in Table C1 in Appendix C. For variable selection, the results of overlapping identifications, RV coefficients, and OOI values are presented in the Supporting Information. The proposed method continues to yield sensible identification and satisfactory prediction.

## 5 | Discussion

In this paper, we have proposed an ordinal sparse neural network for interaction analysis, featuring a novel output function tailored to ordinal outcomes and incorporating a sparse layer for variable selection with the hierarchical structure. The method effectively captures unknown nonlinear relationships and performs variable selection while preserving the hierarchical structure of main effects and interactions. Extensive simulations and real data applications (including G-E and I-E interactions) have demonstrated its competitive performance. Our framework can also be extended to neural network methods based on other ordinal modeling paradigms, such as the continuation ratio model, by appropriately modifying the output layer and loss function. Incorporating other types of interactions (e.g., G-G) is also possible. Future works may also explore theoretical properties and Bayesian formulations for deeper interpretability.

While our method offers flexibility and strong empirical performance, certain limitations may warrant further attention. In particular, modeling higher-order interactions remains challenging due to increased computational complexity [37]. Moreover, the scalability of the proposed framework to ultra-high-dimensional settings warrants further investigation, particularly in cases where the number of covariates greatly exceeds the number of observations. In such scenarios, the computational burden may become substantial, and the stability of feature selection may be affected. Additionally, sensitivity to tuning parameters may warrant further investigation.

## Supplementary Material

Supplementary material

Additional supporting information can be found online in the Supporting Information section. Data S1. Supporting Information.

## Figures and Tables

**TABLE 1 |:** Simulation results for prediction performance in Example 1. In each cell, mean (sd) based on 100 replicates.

		*ρ* = 0.0			*ρ* = 0.25		
*n*	Method	Macro-AUC	RPS	Accuracy	Macro-AUC	RPS	Accuracy
		Balanced proportion					
300	Proposed	0.721 (0.028)	0.372 (0.011)	0.532 (0.033)	0.752 (0.030)	0.359 (0.012)	0.553 (0.039)
	Alt.1	0.660 (0.032)	0.391 (0.011)	0.490 (0.041)	0.694 (0.033)	0.378 (0.012)	0.517 (0.042)
	Alt.2	0.695 (0.029)	0.383 (0.011)	0.505 (0.034)	0.727 (0.033)	0.369 (0.013)	0.530 (0.040)
	Alt.3	0.700 (0.028)	0.394 (0.007)	0.512 (0.033)	0.727 (0.027)	0.385 (0.007)	0.533 (0.032)
	Alt.4	0.689 (0.024)	0.429 (0.002)	0.505 (0.033)	0.719 (0.025)	0.424 (0.003)	0.533 (0.022)
	Alt.6	0.698 (0.024)	0.386 (0.011)	0.523 (0.031)	0.708 (0.027)	0.376 (0.012)	0.536 (0.037)
500	Proposed	0.791 (0.022)	0.344 (0.009)	0.599 (0.030)	0.800 (0.022)	0.339 (0.008)	0.602 (0.031)
	Alt.1	0.713 (0.026)	0.366 (0.010)	0.541 (0.031)	0.744 (0.024)	0.356 (0.009)	0.565 (0.030)
	Alt.2	0.772 (0.022)	0.351 (0.009)	0.583 (0.027)	0.784 (0.022)	0.345 (0.009)	0.585 (0.032)
	Alt.3	0.742 (0.023)	0.381 (0.007)	0.547 (0.027)	0.762 (0.024)	0.372 (0.006)	0.559 (0.032)
	Alt.4	0.744 (0.024)	0.422 (0.002)	0.557 (0.035)	0.766 (0.020)	0.416 (0.002)	0.571 (0.029)
	Alt.6	0.726 (0.019)	0.366 (0.010)	0.558 (0.031)	0.751 (0.017)	0.350 (0.007)	0.579 (0.027)
700	Proposed	0.824 (0.020)	0.331 (0.007)	0.631 (0.029)	0.828 (0.017)	0.329 (0.006)	0.634 (0.028)
	Alt.1	0.753 (0.026)	0.350 (0.009)	0.576 (0.031)	0.773 (0.022)	0.343 (0.008)	0.593 (0.032)
	Alt.2	0.810 (0.021)	0.335 (0.008)	0.615 (0.033)	0.815 (0.019)	0.332 (0.007)	0.618 (0.032)
	Alt.3	0.772 (0.024)	0.374 (0.006)	0.570 (0.026)	0.788 (0.022)	0.366 (0.006)	0.577 (0.028)
	Alt.4	0.766 (0.021)	0.413 (0.001)	0.573 (0.029)	0.785 (0.018)	0.406 (0.003)	0.582 (0.022)
	Alt.6	0.732 (0.017)	0.359 (0.008)	0.566 (0.024)	0.767 (0.019)	0.338 (0.009)	0.593 (0.025)
		Imbalanced proportion					
300	Proposed	0.722 (0.027)	0.334 (0.008)	0.545 (0.033)	0.779 (0.022)	0.315 (0.006)	0.600 (0.034)
	Alt.1	0.632 (0.033)	0.350 (0.009)	0.497 (0.036)	0.680 (0.032)	0.333 (0.009)	0.532 (0.039)
	Alt.2	0.697 (0.029)	0.341 (0.009)	0.527 (0.036)	0.758 (0.024)	0.321 (0.007)	0.583 (0.034)
	Alt.3	0.699 (0.027)	0.353 (0.005)	0.542 (0.031)	0.744 (0.026)	0.342 (0.005)	0.586 (0.034)
	Alt.4	0.689 (0.037)	0.372 (0.002)	0.508 (0.005)	0.728 (0.028)	0.369 (0.002)	0.512 (0.006)
	Alt.6	0.652 (0.030)	0.345 (0.009)	0.517 (0.028)	0.688 (0.027)	0.332 (0.009)	0.553 (0.028)
500	Proposed	0.790 (0.024)	0.314 (0.007)	0.606 (0.029)	0.825 (0.020)	0.300 (0.006)	0.647 (0.031)
	Alt.1	0.678 (0.032)	0.332 (0.009)	0.545 (0.036)	0.737 (0.028)	0.312 (0.008)	0.594 (0.033)
	Alt.2	0.771 (0.023)	0.319 (0.007)	0.589 (0.032)	0.811 (0.021)	0.303 (0.006)	0.632 (0.033)
	Alt.3	0.743 (0.022)	0.344 (0.004)	0.574 (0.030)	0.783 (0.023)	0.333 (0.004)	0.616 (0.034)
	Alt.4	0.761 (0.037)	0.367 (0.002)	0.510 (0.010)	0.778 (0.033)	0.362 (0.002)	0.523 (0.005)
	Alt.6	0.697 (0.022)	0.326 (0.009)	0.542 (0.031)	0.730 (0.017)	0.312 (0.006)	0.587 (0.024)
700	Proposed	0.830 (0.022)	0.304 (0.006)	0.647 (0.034)	0.847 (0.017)	0.294 (0.004)	0.674 (0.026)
	Alt.1	0.722 (0.029)	0.318 (0.009)	0.581 (0.037)	0.762 (0.025)	0.302 (0.007)	0.624 (0.028)
	Alt.2	0.814 (0.023)	0.306 (0.007)	0.632 (0.034)	0.836 (0.018)	0.296 (0.005)	0.659 (0.028)
	Alt.3	0.775 (0.024)	0.340 (0.004)	0.601 (0.031)	0.806 (0.020)	0.330 (0.004)	0.636 (0.027)
	Alt.4	0.767 (0.015)	0.360 (0.001)	0.523 (0.007)	0.795 (0.024)	0.354 (0.003)	0.561 (0.010)
	Alt.6	0.707 (0.017)	0.321 (0.007)	0.553 (0.028)	0.745 (0.018)	0.303 (0.006)	0.600 (0.027)

**TABLE 2 |:** G-E interaction analysis: Identified genes and interactions.

	Interactions			
Genes	Diagnosis age	ER status	PR status	HER2
MOXD2	*			
EFCAB8				
GGACT				
AAAS				
AADACL4	*			
ABCA5	*		*	
ABCA6			*	*
ABCB1				
ABCC5	*			
ABCG1		*	*	
ABHD1	*			
ABHD6	*			
ABI1	*			
ABI3BP	*		*	
ABRA				*
ABT1				
ACACB		*		
ACADS	*		*	
ACAN	*			
ASIC5	*			
ACCSL	*			
ACE2	*			
ACMSD	*			
ACO2				
ACOT8	*			
ACPP	*			*
ACRBP				
ACTG2	*			

**TABLE 3 |:** G-E interaction analysis: Overlapping identifications (and RV-coefficients) of the proposed and alternative methods.

Genes	Proposed	Alt.1	Alt.2	Alt.4	Alt.5

Proposed	28 (1.00)	17 (0.79)	23 (0.92)	13 (0.71)	2 (0.45)
Alt.1		21 (1.00)	18 (0.86)	8 (0.64)	1 (0.55)
Alt.2			25 (1.00)	13 (0.72)	2 (0.49)
Alt.4				30 (1.00)	2 (0.53)
Alt.5					20 (1.00)

Interactions	Proposed	Alt.1	Alt.2	Alt.4	Alt.5

Proposed	27 (1.00)	16 (0.77)	20 (0.86)	7 (0.50)	0 (0.25)
Alt.1		26 (1.00)	19 (0.87)	6 (0.51)	0 (0.26)
Alt.2			26 (1.00)	6 (0.47)	0 (0.27)
Alt.4				26 (1.00)	1 (0.26)
Alt.5					21 (1.00)

## Data Availability

The Cancer Genome Atlas (TCGA) data that support the findings in this paper are publicly available at the National Cancer Institute Genomic Data Commons Data Portal (https://portal.gdc.cancer.gov/). The data that support the findings of this study are available in The Cancer Genome Atlas at: https://www.cancer.gov/ccg/research/genome-sequencing/tcga. These data were derived from the following resources available in the public domain: The Cancer Genome Atlas, https://portal.gdc.cancer.gov.

## Appendix A Additional Definitions

We provide some additional definitions in this section.

- *Logistic distribution:* A random variable *ξ* follows a standard Logistic distribution *ℒ*(*μ*, *σ*) if and only if the cumulative distribution function is
  (A1)
  $$F_{\xi }(x)=\frac{1}{1+exp\left(-\frac{x-\mu }{\sigma }\right)},\;x\in \mathbb{R},$$
  where μ and σ are the location parameter and scale parameter, respectively.
- *Macro-AUC:* Macro-AUC computes the average of AUC (Area Under the ROC Curve) scores for each class independently in a multiclass setting, treating all classes equally regardless of class imbalance. The macro-AUC is defined as
  $$AUC_{\mathrm{macro}}=\frac{1}{K}\sum_{k=1}^{K}AUC_{k},$$
  where K denotes the number of classes, and $AUC_{k}$ is the AUC score for class k (vs. rest).
- *RPS:* RPS compares the stepwise cumulative probabilities (P(Y≤k) for each ordinal class) generated by the model against the observed cumulative distribution (1 if Y≤k,0 otherwise). The definition of RPS is given by:
  $$RPS=\frac{1}{n}\sum_{i=1}^{n}\sum_{k=1}^{K}{\left[{\text{Π}}_{i}(k)-1\left(Y_{i}\leq k\right)\right]}^{2},$$
  where $\Pi_{i}(k)$ is the predicted probability of observation i belonging to class k=1,…,K, and 1(⋅) denotes the indicator function.

## Appendix B Additional Simulation Settings

We describe more simulation settings in this section. Additional simulation results are summarized in the Supporting Information.

### Example B1 (Linear setting).

We consider a linear setting, and the latent variable is generated by:

$$u_{i}=\sum_{j=1}^{15}\omega_{j}x_{ij}+\sum_{j=1}^{15}c_{j1}x_{ij}z_{i1}+\sum_{d=1}^{5}s_{d}z_{id}+\epsilon_{i},$$

where coefficients $\omega_{j},c_{j1}$ and $s_{d}$ are from 𝒱(0.6, 0.9), and error term $\epsilon_{i}$ follows the standard Logistic distribution ℒ(0, 1).

### Example B2 (Nonlinear setting).

We set another nonlinear setting in that

$$u_{i}=-2(\omega_{0}+\sum_{j=1}^{15}\omega_{j}x_{\mathrm{ij}}+\sum_{j=1}^{15}c_{\mathrm{j1}}x_{\mathrm{ij}}z_{\mathrm{i1}}+\sum_{d=1}^{5}s_{d}z_{\mathrm{id}}{)}^{2}+\epsilon_{i},$$

where intercept $\omega_{0}$ is 10. The coefficients are the same as the aforementioned settings. Error terms $\epsilon_{i}$ is from normal distribution 𝒩(0, 1). The proportions of labels are controlled by threshold parameters $a_{0},\ldots ,a_{3}$.

## Appendix C Additional Results for Real Data Analysis

**TABLE C1 |:** Prediction results averaged over 100 random data splittings.

Method	BRCA				SKCM		
	Macro-AUC	RPS	Accuracy		Macro-AUC	RPS	Accuracy
Proposed	0.622 (0.030)	0.413 (0.010)	0.535 (0.032)		0.553 (0.065)	0.472 (0.015)	0.426 (0.076)
Alt.1	0.584 (0.029)	0.434 (0.014)	0.472 (0.035)		0.428 (0.074)	0.483 (0.030)	0.416 (0.078)
Alt.2	0.615 (0.029)	0.424 (0.011)	0.529 (0.033)		0.421 (0.071)	0.481 (0.021)	0.410 (0.073)
Alt.3	0.604 (0.028)	0.428 (0.013)	0.491 (0.033)		0.427 (0.069)	0.485 (0.027)	0.414 (0.060)
Alt.4	0.591 (0.028)	0.431 (0.014)	0.487 (0.035)		0.414 (0.074)	0.480 (0.015)	0.414 (0.060)
Alt.6	0.603 (0.028)	0.412 (0.017)	0.498 (0.035)		0.582 (0.072)	0.478 (0.033)	0.423 (0.077)

**TABLE C2 |:** G-E interaction analysis: OOI values of selected genes and interactions based on 100 random splittings using the proposed method.

Genes	OOI of main effects	OOI of interactions			
		Diagnosis age	ER status	PR status	HER2
MOXD2	0.98	0.97			
LOC155060	0.43			0.43	
EZHIP	0.45				
EFCAB8	0.76	0.66			
AAAS	0.95	0.43			
ABCA6	0.84			0.53	
PXE	0.41				
ABCG1	0.72		0.72		0.65
ABHD1	0.62				
ABHD6	0.51				
ABI1	0.64	0.60			
ABI3BP	0.59	0.57			
ABT1	0.53				
ACACB	0.51		0.49		
ACADS	0.61	0.59		0.44	
ACAN	0.95	0.85			
ASIC5	0.98	0.98			0.47
ACCSL	0.81	0.79			
ACE2	1.00	1.00			
ACOT8	0.99	0.93	0.44		
ACPP	0.69	0.67			0.46
ACRBP	0.80	0.47			
ACSS2	0.75	0.75			
ACTG2	0.47	0.43			

## References

[1] Herrera-Luis E, Benke K, Volk H, Ladd-Acosta C, and Wojcik GL, “Gene–Environment Interactions in Human Health,” Nature Reviews Genetics 25, no. 11 (2024): 768–817. 10.1038/s41576-024-00731-z PMC12288441 38806721

[2] Xu Y, Zhong T, Wu M, and Ma S, “Histopathological Imaging–Environment Interactions in Cancer Modeling,” Cancers 11, no. 4 (2019): 579. 31022926 10.3390/cancers11040579 PMC6520737

[3] Zhang S, Xue Y, Zhang Q, Ma C, Wu M, and Ma S, “Identification of Gene–Environment Interactions With Marginal Penalization,” Genetic Epidemiology 44, no. 2 (2020): 159–196. 31724772 10.1002/gepi.22270 PMC7028443

[4] Liu J, Huang J, Zhang Y, “Identification of Gene–Environment Interactions in Cancer Studies Using Penalization,” Genomics 102, no. 4 (2013): 189–194. 23994599 10.1016/j.ygeno.2013.08.006 PMC3869641

[5] Hao N and Zhang HH, “Interaction Screening for Ultrahigh-Dimensional Data,” Journal of the American Statistical Association 109, no. 507 (2014): 1285–1301. 25386043 10.1080/01621459.2014.881741 PMC4224119

[6] Chaste P and Leboyer M, “Autism Risk Factors: Genes, Environment, and Gene-Environment Interactions,” Dialogues in Clinical Neuroscience 14, no. 3 (2012): 281–292. 23226953 10.31887/DCNS.2012.14.3/pchaste PMC3513682

[7] Lim M and Hastie T, “Learning Interactions via Hierarchical Group-Lasso Regularization,” Journal of Computational and Graphical Statistics 24, no. 3 (2015): 627–654. 26759522 10.1080/10618600.2014.938812 PMC4706754

[8] Im Y, Huang Y, Tan A, and Ma S, “Bayesian Finite Mixture of Regression Analysis for Cancer Based on Histopathological Imaging–Environment Interactions,” Biostatistics 24, no. 2 (2023): 425–442. 37057611 10.1093/biostatistics/kxab038 PMC10102889

[9] Lee J, “Cumulative Logit Modelling for Ordinal Response Variables: Applications to Biomedical Research,” Bioinformatics 8, no. 6 (1992): 555–562. 10.1093/bioinformatics/8.6.555 1468011

[10] Archer KJ and Williams AA, “L 1 Penalized Continuation Ratio Models for Ordinal Response Prediction Using High-Dimensional Datasets,” Statistics in Medicine 31, no. 14 (2012): 1464–1474. 22359384 10.1002/sim.4484 PMC3718008

[11] Baccianella S, Esuli A, and Sebastiani F, “Feature Selection for Ordinal Regression,” in Proceedings of the 2010 ACM Symposium on Applied Computing (ACM, 2010), 1748–1754.

[12] Gentry AE, Jackson-Cook CK, Lyon DE, and Archer KJ, “Penalized Ordinal Regression Methods for Predicting Stage of Cancer in High-Dimensional Covariate Spaces,” Cancer Informatics 14 (2015): CIN-S17277. 10.4137/CIN.S17277 PMC4447150 26052223

[13] Hastie T and Tibshirani R, “Non-Parametric Logistic and Proportional Odds Regression,” Journal of the Royal Statistical Society: Series C: Applied Statistics 36, no. 3 (1987): 260–276.

[14] DeYoreo M and Kottas A, “Bayesian Nonparametric Modeling for Multivariate Ordinal Regression,” Journal of Computational and Graphical Statistics 27, no. 1 (2018): 71–84.

[15] Bao J and Hanson TE, “Bayesian Nonparametric Multivariate Ordinal Regression,” Canadian Journal of Statistics 43, no. 3 (2015): 337–357.

[16] Bien J, Taylor J, and Tibshirani R, “A Lasso for Hierarchical Interactions,” Annals of Statistics 41, no. 3 (2013): 1111–1141. 26257447 10.1214/13-AOS1096 PMC4527358

[17] Min S, Lee B, and Yoon S, “Deep Learning in Bioinformatics,” Briefings in Bioinformatics 18, no. 5 (2017): 851–869. 27473064 10.1093/bib/bbw068

[18] Eraslan G, Avsec Ž, Gagneur J, and Theis FJ, “Deep Learning: New Computational Modelling Techniques for Genomics,” Nature Reviews Genetics 20, no. 7 (2019): 389–403. 10.1038/s41576-019-0122-6 30971806

[19] Dinh VC and Ho LS, “Consistent Feature Selection for Analytic Deep Neural Networks,” Advances in Neural Information Processing Systems 33 (2020): 2420–2431.

[20] Chen Y, Gao Q, Liang F, and Wang X, “Nonlinear Variable Selection via Deep Neural Networks,” Journal of Computational and Graphical Statistics 30, no. 2 (2021): 484–492.

[21] Rask-Andersen M, Karlsson T, Ek WE, and Johansson Å, “Gene-Environment Interaction Study for BMI Reveals Interactions Between Genetic Factors and Physical Activity, Alcohol Consumption and Socioeconomic Status,” PLoS Genetics 13, no. 9 (2017): e1006977. 28873402 10.1371/journal.pgen.1006977 PMC5600404

[22] Jiao Y, Shen G, Lin Y, and Huang J, “Deep Nonparametric Regression on Approximate Manifolds: Nonasymptotic Error Bounds With Polynomial Prefactors,” Annals of Statistics 51, no. 2 (2023): 691–716.

[23] Zhang CH, “Nearly Unbiased Variable Selection Under Minimax Concave Penalty,” Annals of Statistics 38, no. 2 (2010): 894–942.

[24] Prince SJ, Understanding Deep Learning (MIT Press, 2023).

[25] Fan J and Li R, “Variable Selection via Nonconcave Penalized Likelihood and Its Oracle Properties,” Journal of the American Statistical Association 96, no. 456 (2001): 1348–1360.

[26] Li Y, Chen CY, and Wasserman WW, “Deep Feature Selection: Theory and Application to Identify Enhancers and Promoters,” Journal of Computational Biology 23, no. 5 (2016): 322–336. 26799292 10.1089/cmb.2015.0189

[27] Wu S, Xu Y, Zhang Q, and Ma S, “Gene–Environment Interaction Analysis via Deep Learning,” Genetic Epidemiology 47, no. 3 (2023): 261–286. 36807383 10.1002/gepi.22518 PMC10244912

[28] Lechner M and Okasa G, “Random Forest Estimation of the Ordered Choice Model,” Empirical Economics 68, no. 1 (2025): 1–106.

[29] Zhang ML and Zhou ZH, “A Review on Multi-Label Learning Algorithms,” IEEE Transactions on Knowledge and Data Engineering 26, no. 8 (2013): 1819–1837.

[30] Murphy AH, “The Ranked Probability Score and the Probability Score: A Comparison,” Monthly Weather Review 98, no. 12 (1970): 917–924.

[31] Xiong X, Zheng LW, Ding Y, “Breast Cancer: Pathogenesis and Treatments,” Signal Transduction and Targeted Therapy 10, no. 1 (2025): 49. 39966355 10.1038/s41392-024-02108-4 PMC11836418

[32] Wang F, Liang D, Li Y, and Ma S, “Prior Information-Assisted Integrative Analysis of Multiple Datasets,” Bioinformatics 39, no. 8 (2023): btad452. 37490475 10.1093/bioinformatics/btad452 PMC10400378

[33] Giaquinto AN, Sung H, Miller KD, “Breast Cancer Statistics, 2022,” CA: A Cancer Journal for Clinicians 72, no. 6 (2022): 524–541. 36190501 10.3322/caac.21754

[34] Smilde AK, Kiers HA, Bijlsma S, Rubingh C, and Van Erk M, “Matrix Correlations for High-Dimensional Data: The Modified RV-Coefficient,” Bioinformatics 25, no. 3 (2009): 401–405. 19073588 10.1093/bioinformatics/btn634

[35] Gurcan MN, Boucheron LE, Can A, Madabhushi A, Rajpoot NM, and Yener B, “Histopathological Image Analysis: A Review,” IEEE Reviews in Biomedical Engineering 2 (2009): 147–171. 20671804 10.1109/RBME.2009.2034865 PMC2910932

[36] Luo X, Zang X, Yang L, “Comprehensive Computational Pathological Image Analysis Predicts Lung Cancer Prognosis,” Journal of Thoracic Oncology 12, no. 3 (2017): 501–509. 27826035 10.1016/j.jtho.2016.10.017 PMC5462113

[37] Tsang M, Liu H, Purushotham S, Murali P, and Liu Y, “Neural Interaction Transparency (Nit): Disentangling Learned Interactions for Improved Interpretability,” Advances in Neural Information Processing Systems 31 (2018).

