# Supplementary material for: Ordinal Sparse Neural Networks for Modeling Gene- and Imaging-Environment Interactions
Source: Stat Med. Author manuscript; Available in PMC 2026 Jun 3. (PMC13231275; doi:10.1002/sim.70302)
Supplement: Supplementary material [file NIHMS2172199-supplement-Supplementary_material.pdf]

Supplementary Materials for "Ordinal Sparse Neural Networks for Modeling Gene- and Imaging-Environment Interactions" by Jiajing Xue, Yaqing Xu, Jingmao Li, Shuangge Ma and Kuangnan Fang

A Additional simulation results

Table A1: Simulation results for prediction performance in Example 1 with weak interaction signals. In each cell, mean (sd) based on 100 replicates.

n	Method	Balanced label			Imbalanced label		
		macro-AUC	RPS	accuracy	macro-AUC	RPS	accuracy
300	Proposed	0.725 (0.028)	0.373 (0.011)	0.525 (0.037)	0.693 (0.030)	0.334 (0.008)	0.548 (0.033)
	Alt.1	0.664 (0.031)	0.391 (0.011)	0.491 (0.037)	0.622 (0.034)	0.353 (0.009)	0.492 (0.036)
	Alt.2	0.680 (0.031)	0.392 (0.012)	0.484 (0.038)	0.653 (0.032)	0.344 (0.009)	0.525 (0.034)
	Alt.3	0.632 (0.025)	0.416 (0.006)	0.451 (0.033)	0.617 (0.026)	0.364 (0.005)	0.494 (0.026)
	Alt.4	0.686 (0.027)	0.390 (0.009)	0.499 (0.034)	0.682 (0.024)	0.342 (0.007)	0.536 (0.030)
	Alt.6	0.653 (0.028)	0.409 (0.010)	0.480 (0.035)	0.600 (0.026)	0.364 (0.007)	0.487 (0.026)
500	Proposed	0.774 (0.021)	0.349 (0.009)	0.586 (0.033)	0.752 (0.025)	0.316 (0.008)	0.600 (0.033)
	Alt.1	0.713 (0.024)	0.368 (0.009)	0.535 (0.031)	0.665 (0.034)	0.338 (0.009)	0.521 (0.036)
	Alt.2	0.745 (0.023)	0.362 (0.010)	0.552 (0.031)	0.723 (0.029)	0.323 (0.009)	0.576 (0.033)
	Alt.3	0.664 (0.024)	0.407 (0.006)	0.483 (0.032)	0.646 (0.024)	0.359 (0.004)	0.506 (0.029)
	Alt.4	0.724 (0.021)	0.377 (0.007)	0.537 (0.029)	0.716 (0.022)	0.334 (0.006)	0.565 (0.027)
	Alt.6	0.691 (0.023)	0.392 (0.008)	0.516 (0.031)	0.638 (0.025)	0.352 (0.007)	0.500 (0.024)
700	Proposed	0.802 (0.019)	0.337 (0.008)	0.617 (0.030)	0.784 (0.021)	0.306 (0.007)	0.623 (0.031)
	Alt.1	0.757 (0.021)	0.355 (0.008)	0.575 (0.028)	0.713 (0.031)	0.327 (0.008)	0.568 (0.034)
	Alt.2	0.777 (0.023)	0.346 (0.009)	0.589 (0.032)	0.763 (0.023)	0.311 (0.008)	0.604 (0.030)
	Alt.3	0.682 (0.025)	0.404 (0.005)	0.501 (0.034)	0.662 (0.023)	0.358 (0.004)	0.521 (0.028)
	Alt.4	0.741 (0.021)	0.371 (0.007)	0.550 (0.032)	0.732 (0.022)	0.330 (0.005)	0.575 (0.029)
	Alt.6	0.707 (0.022)	0.383 (0.008)	0.532 (0.032)	0.648 (0.021)	0.348 (0.006)	0.506 (0.025)

Table A2: Simulation results for prediction performance in Example 2. In each cell, mean (sd) based on 100 replicates.

n	Method	$\rho = 0.0$			$\rho = 0.25$		
		macro-AUC	RPS	accuracy	macro-AUC	RPS	accuracy
				Balanced proportion			
300	Proposed	0.726 (0.026)	0.371 (0.010)	0.536 (0.032)	0.758 (0.026)	0.358 (0.010)	0.561 (0.034)
	Alt.1	0.663 (0.029)	0.391 (0.010)	0.490 (0.034)	0.697 (0.032)	0.377 (0.011)	0.518 (0.039)
	Alt.2	0.697 (0.028)	0.383 (0.010)	0.506 (0.037)	0.734 (0.028)	0.367 (0.011)	0.540 (0.038)
	Alt.3	0.701 (0.028)	0.394 (0.007)	0.511 (0.030)	0.729 (0.028)	0.385 (0.007)	0.535 (0.036)
	Alt.4	0.684 (0.033)	0.428 (0.003)	0.486 (0.032)	0.712 (0.030)	0.426 (0.002)	0.528 (0.034)
	Alt.6	0.702 (0.025)	0.386 (0.011)	0.528 (0.034)	0.713 (0.026)	0.376 (0.012)	0.542 (0.030)
500	Proposed	0.806 (0.021)	0.339 (0.008)	0.618 (0.029)	0.815 (0.020)	0.334 (0.007)	0.619 (0.030)
	Alt.1	0.724 (0.028)	0.363 (0.011)	0.554 (0.033)	0.752 (0.025)	0.352 (0.009)	0.574 (0.033)
	Alt.2	0.784 (0.022)	0.347 (0.008)	0.596 (0.031)	0.798 (0.021)	0.340 (0.008)	0.603 (0.031)
	Alt.3	0.750 (0.024)	0.379 (0.007)	0.555 (0.032)	0.771 (0.022)	0.371 (0.006)	0.565 (0.027)
	Alt.4	0.765 (0.021)	0.421 (0.002)	0.568 (0.031)	0.774 (0.022)	0.416 (0.002)	0.592 (0.028)
	Alt.6	0.736 (0.022)	0.364 (0.011)	0.569 (0.030)	0.756 (0.019)	0.350 (0.009)	0.592 (0.027)
700	Proposed	0.845 (0.018)	0.325 (0.007)	0.651 (0.027)	0.841 (0.015)	0.325 (0.005)	0.647 (0.027)
	Alt.1	0.769 (0.025)	0.344 (0.009)	0.592 (0.032)	0.782 (0.021)	0.339 (0.007)	0.601 (0.031)
	Alt.2	0.830 (0.020)	0.329 (0.008)	0.636 (0.030)	0.828 (0.016)	0.328 (0.006)	0.634 (0.029)
	Alt.3	0.781 (0.024)	0.372 (0.006)	0.575 (0.027)	0.796 (0.018)	0.364 (0.005)	0.584 (0.026)
	Alt.4	0.776 (0.027)	0.411 (0.002)	0.583 (0.037)	0.798 (0.016)	0.405 (0.002)	0.606 (0.025)
	Alt.6	0.748 (0.015)	0.355 (0.008)	0.581 (0.029)	0.766 (0.017)	0.341 (0.008)	0.604 (0.024)
				Imbalanced proportion			
300	Proposed	0.758 (0.029)	0.324 (0.008)	0.589 (0.037)	0.801 (0.023)	0.308 (0.007)	0.627 (0.037)
	Alt.1	0.656 (0.032)	0.343 (0.009)	0.517 (0.035)	0.701 (0.031)	0.326 (0.009)	0.559 (0.037)
	Alt.2	0.730 (0.030)	0.332 (0.009)	0.564 (0.037)	0.777 (0.024)	0.315 (0.007)	0.603 (0.037)
	Alt.3	0.722 (0.028)	0.349 (0.005)	0.571 (0.034)	0.759 (0.023)	0.339 (0.005)	0.601 (0.031)
	Alt.4	0.708 (0.028)	0.371 (0.002)	0.509 (0.008)	0.757 (0.023)	0.367 (0.002)	0.516 (0.007)
	Alt.6	0.711 (0.027)	0.329 (0.010)	0.560 (0.030)	0.699 (0.027)	0.327 (0.009)	0.558 (0.027)
500	Proposed	0.838 (0.023)	0.301 (0.006)	0.662 (0.035)	0.854 (0.018)	0.291 (0.006)	0.685 (0.032)
	Alt.1	0.716 (0.033)	0.320 (0.009)	0.583 (0.037)	0.766 (0.026)	0.303 (0.008)	0.628 (0.031)
	Alt.2	0.815 (0.022)	0.306 (0.007)	0.640 (0.035)	0.838 (0.020)	0.295 (0.006)	0.668 (0.030)
	Alt.3	0.774 (0.025)	0.339 (0.004)	0.613 (0.033)	0.803 (0.020)	0.329 (0.004)	0.640 (0.028)
	Alt.4	0.775 (0.029)	0.365 (0.002)	0.512 (0.008)	0.804 (0.020)	0.360 (0.002)	0.533 (0.012)
	Alt.6	0.747 (0.021)	0.310 (0.009)	0.587 (0.028)	0.742 (0.017)	0.305 (0.007)	0.601 (0.027)
700	Proposed	0.876 (0.018)	0.290 (0.005)	0.705 (0.033)	0.877 (0.014)	0.285 (0.004)	0.713 (0.027)
	Alt.1	0.762 (0.029)	0.304 (0.008)	0.631 (0.033)	0.795 (0.024)	0.292 (0.006)	0.661 (0.026)
	Alt.2	0.860 (0.019)	0.293 (0.006)	0.688 (0.032)	0.865 (0.016)	0.286 (0.004)	0.698 (0.029)
	Alt.3	0.809 (0.024)	0.334 (0.004)	0.645 (0.032)	0.829 (0.019)	0.325 (0.004)	0.665 (0.030)
	Alt.4	0.807 (0.011)	0.358 (0.001)	0.533 (0.013)	0.825 (0.018)	0.351 (0.002)	0.568 (0.017)
	Alt.6	0.762 (0.016)	0.302 (0.007)	0.605 (0.022)	0.755 (0.015)	0.296 (0.006)	0.610 (0.025)

Table A3: Simulation results for prediction performance in Example 3. In each cell, mean (sd) based on 100 replicates.

n	Method	$\rho = 0.0$			$\rho = 0.25$		
		macro-AUC	RPS	accuracy	macro-AUC	RPS	accuracy
				Balanced proportion			
300	Proposed	0.791 (0.027)	0.347 (0.010)	0.606 (0.032)	0.808 (0.026)	0.340 (0.010)	0.621 (0.033)
	Alt.1	0.710 (0.030)	0.372 (0.011)	0.538 (0.036)	0.732 (0.029)	0.362 (0.010)	0.560 (0.034)
	Alt.2	0.753 (0.029)	0.361 (0.011)	0.567 (0.037)	0.776 (0.027)	0.353 (0.010)	0.588 (0.033)
	Alt.3	0.750 (0.027)	0.382 (0.007)	0.558 (0.033)	0.772 (0.027)	0.375 (0.007)	0.581 (0.033)
	Alt.4	0.737 (0.020)	0.425 (0.001)	0.551 (0.024)	0.766 (0.022)	0.421 (0.002)	0.583 (0.032)
	Alt.6	0.781 (0.028)	0.343 (0.014)	0.620 (0.039)	0.802 (0.019)	0.332 (0.011)	0.637 (0.028)
500	Proposed	0.882 (0.024)	0.310 (0.009)	0.699 (0.035)	0.869 (0.025)	0.315 (0.010)	0.687 (0.035)
	Alt.1	0.790 (0.027)	0.337 (0.011)	0.625 (0.035)	0.791 (0.029)	0.335 (0.011)	0.623 (0.038)
	Alt.2	0.854 (0.026)	0.319 (0.010)	0.670 (0.038)	0.844 (0.024)	0.323 (0.010)	0.659 (0.037)
	Alt.3	0.805 (0.024)	0.364 (0.007)	0.606 (0.032)	0.817 (0.021)	0.359 (0.006)	0.617 (0.027)
	Alt.4	0.801 (0.016)	0.417 (0.002)	0.619 (0.018)	0.816 (0.014)	0.412 (0.002)	0.641 (0.030)
	Alt.6	0.810 (0.020)	0.318 (0.011)	0.657 (0.033)	0.836 (0.015)	0.304 (0.008)	0.677 (0.021)
700	Proposed	0.925 (0.017)	0.292 (0.007)	0.754 (0.029)	0.905 (0.017)	0.301 (0.007)	0.735 (0.030)
	Alt.1	0.846 (0.025)	0.313 (0.010)	0.683 (0.033)	0.833 (0.023)	0.318 (0.009)	0.667 (0.032)
	Alt.2	0.910 (0.017)	0.297 (0.007)	0.737 (0.030)	0.888 (0.018)	0.306 (0.007)	0.713 (0.030)
	Alt.3	0.841 (0.021)	0.354 (0.006)	0.635 (0.027)	0.844 (0.017)	0.351 (0.005)	0.633 (0.023)
	Alt.4	0.806 (0.013)	0.407 (0.001)	0.614 (0.032)	0.849 (0.019)	0.399 (0.003)	0.668 (0.029)
	Alt.6	0.824 (0.020)	0.304 (0.011)	0.673 (0.029)	0.851 (0.012)	0.290 (0.007)	0.694 (0.022)
				Imbalanced proportion			
300	Proposed	0.811 (0.029)	0.309 (0.009)	0.643 (0.036)	0.835 (0.027)	0.300 (0.008)	0.675 (0.037)
	Alt.1	0.698 (0.031)	0.329 (0.009)	0.563 (0.037)	0.732 (0.032)	0.316 (0.010)	0.599 (0.037)
	Alt.2	0.776 (0.031)	0.318 (0.009)	0.607 (0.038)	0.807 (0.029)	0.307 (0.009)	0.649 (0.039)
	Alt.3	0.756 (0.027)	0.342 (0.005)	0.606 (0.034)	0.787 (0.027)	0.335 (0.005)	0.640 (0.034)
	Alt.4	0.734 (0.017)	0.369 (0.001)	0.509 (0.004)	0.779 (0.023)	0.366 (0.002)	0.516 (0.010)
	Alt.6	0.785 (0.023)	0.297 (0.010)	0.617 (0.030)	0.794 (0.018)	0.295 (0.009)	0.632 (0.027)
500	Proposed	0.896 (0.020)	0.283 (0.006)	0.729 (0.033)	0.888 (0.020)	0.282 (0.006)	0.739 (0.029)
	Alt.1	0.779 (0.029)	0.300 (0.009)	0.649 (0.034)	0.794 (0.029)	0.292 (0.009)	0.676 (0.032)
	Alt.2	0.870 (0.022)	0.289 (0.007)	0.703 (0.033)	0.868 (0.021)	0.287 (0.007)	0.716 (0.030)
	Alt.3	0.812 (0.021)	0.331 (0.004)	0.651 (0.032)	0.834 (0.019)	0.325 (0.004)	0.689 (0.027)
	Alt.4	0.800 (0.014)	0.363 (0.001)	0.515 (0.006)	0.828 (0.020)	0.359 (0.001)	0.533 (0.014)
	Alt.6	0.808 (0.016)	0.280 (0.008)	0.644 (0.027)	0.824 (0.014)	0.273 (0.007)	0.666 (0.024)
700	Proposed	0.930 (0.013)	0.272 (0.005)	0.773 (0.030)	0.919 (0.014)	0.274 (0.005)	0.780 (0.027)
	Alt.1	0.831 (0.027)	0.281 (0.008)	0.708 (0.033)	0.830 (0.024)	0.279 (0.007)	0.714 (0.032)
	Alt.2	0.915 (0.014)	0.275 (0.005)	0.756 (0.031)	0.904 (0.015)	0.276 (0.005)	0.758 (0.030)
	Alt.3	0.849 (0.020)	0.325 (0.004)	0.688 (0.033)	0.865 (0.017)	0.319 (0.004)	0.719 (0.030)
	Alt.4	0.819 (0.012)	0.355 (0.001)	0.527 (0.011)	0.874 (0.021)	0.348 (0.002)	0.579 (0.015)
	Alt.6	0.823 (0.015)	0.270 (0.007)	0.660 (0.026)	0.838 (0.012)	0.262 (0.006)	0.678 (0.021)

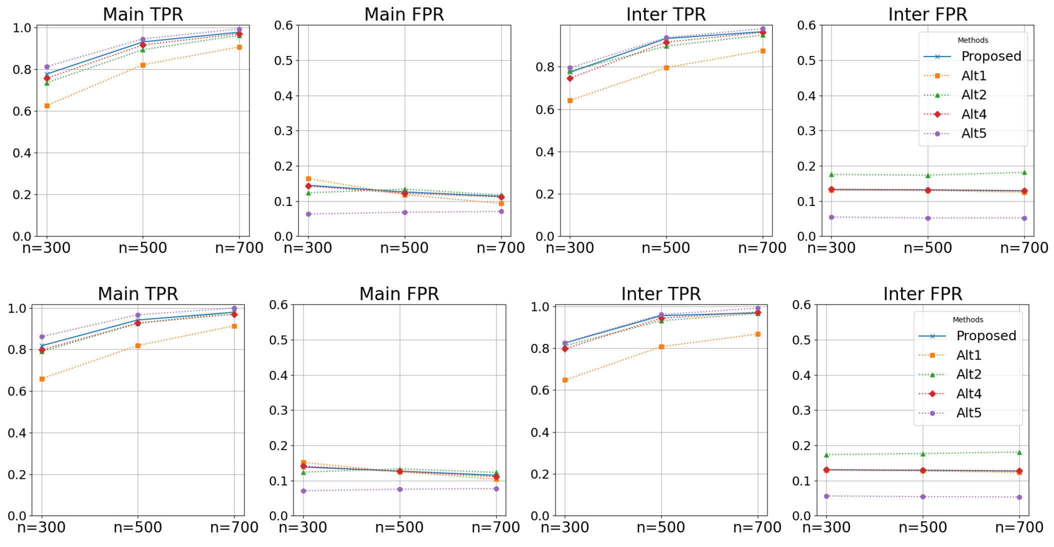

Figure A1: Simulation results for variable selection in Example 1 with AR(0.25). The first and second rows correspond to response class proportions of 1 : 1 : 1 and 1 : 2 : 1, respectively. “Main” refers to main effects, and “Inter” to interactions.

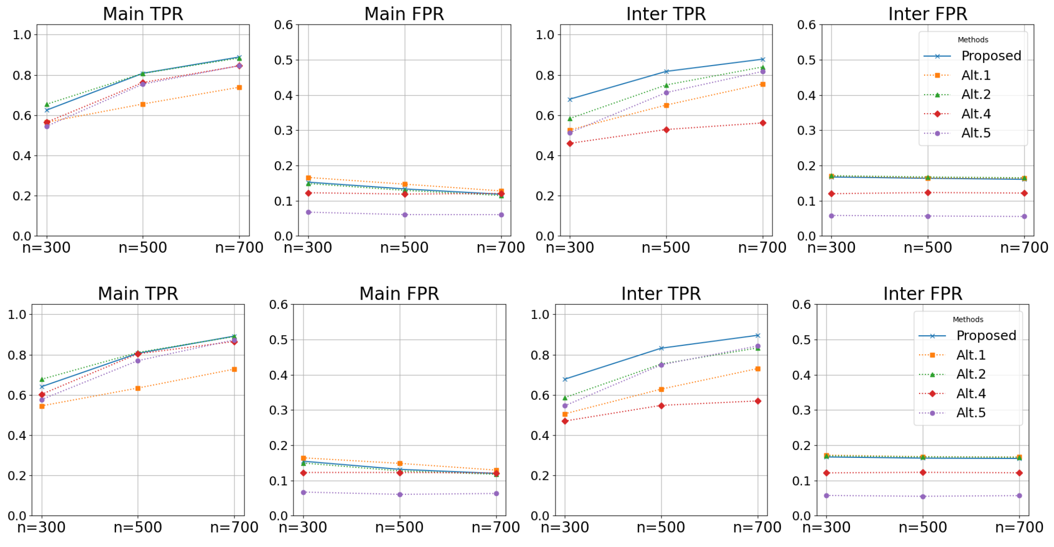

Figure A2: Simulation results for variable selection in Example 1 with weak interaction signals. The first and second rows correspond to response class proportions of 1 : 1 : 1 and 1 : 2 : 1, respectively. “Main” refers to main effects, and “Inter” to interactions.

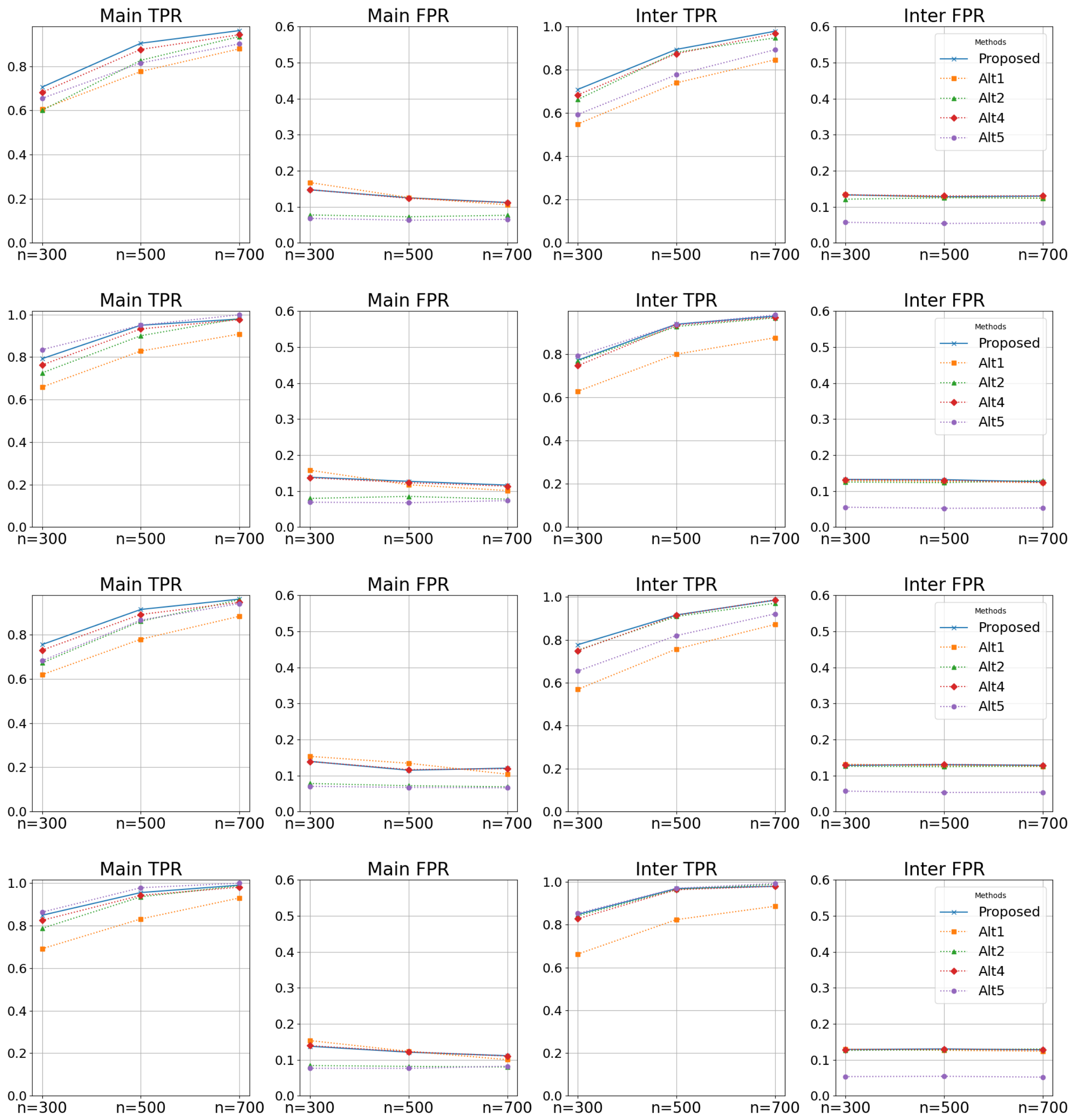

Figure A3: Simulation results for variable selection in Example 2. Panels are ordered from top to bottom as follows: (AR(0.0), 1:1:1), (AR(0.25), 1:1:1), (AR(0.0), 1:2:1), and (AR(0.25), 1:2:1), where each pair denotes (correlation structure, response class proportion).

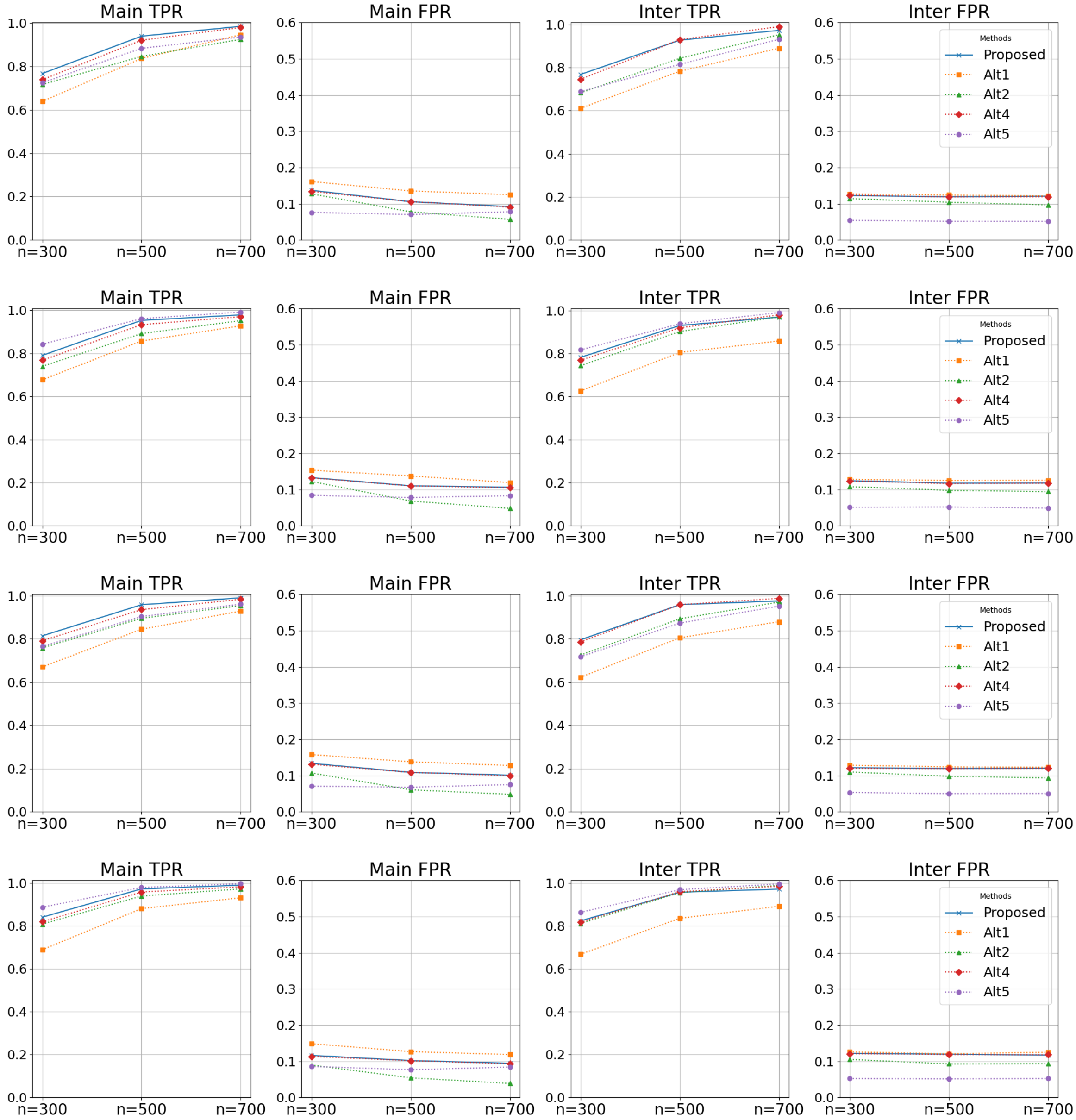

Figure A4: Simulation results for variable selection in Example 3. Panels are ordered from top to bottom as follows: (AR(0.0), 1:1:1), (AR(0.25), 1:1:1), (AR(0.0), 1:2:1), and (AR(0.25), 1:2:1), where each pair denotes (correlation structure, response class proportion).

B Additional results for real data analysis

Table B4: I-E interaction analysis: identified imaging features and interactions.

Imaging features	Interactions				
	Diagnosis age	Gender	AS	FG	MC
AreaShape_Zernike_8_6				*	
AreaShape_Zernike_8_8					
AreaShape_Extent					
AreaShape_BoundingBoxMinimum_Y				*	
AreaShape_Orientation			*	*	*
Count_identifyhemaprimarynuclei					
Granularity_1_ImageAfterMath	*				
Granularity_8_ImageAfterMath					
Granularity_15_ImageAfterMath	*	*			
Granularity_3_ImageAfterMath.1	*				
Granularity_5_ImageAfterMath.1	*	*			
Granularity_8_ImageAfterMath.1		*			
Granularity_9_ImageAfterMath.1	*		*		*
Granularity_10_ImageAfterMath.1		*	*	*	
Granularity_16_ImageAfterMath.1	*	*	*		
Texture_Correlation_maskosingray_3_01_256	*				
Texture_Correlation_ImageAfterMath_3_02_256	*	*			
Texture_Entropy_ImageAfterMath_3_01_256					
Texture_SumAverage_ImageAfterMath_3_00_256	*				
Texture_SumAverage_ImageAfterMath_3_01_256	*		*		
Texture_SumEntropy_ImageAfterMath_3_00_256					
Threshold_SumOfEntropies_Identifyeosinprimarycytoplasm	*		*		*
Threshold_SumOfEntropies_identifyhemaprimarynuclei	*	*			
Threshold_WeightedVariance_Identifyeosinprimarycytoplasm		*			
Threshold_WeightedVariance_identifytissueregion		*			

Table B5: I-E interaction analysis: overlapping identifications (and RV-coefficients) of the proposed and alternative methods.

Imaging features	Proposed	Alt.1	Alt.2	Alt.4	Alt.5
Proposed	25(1.00)	14(0.89)	20(0.95)	13(0.88)	3(0.55)
Alt.1		22(1.00)	17(0.95)	13(0.91)	2(0.61)
Alt.2			26(1.00)	19(0.95)	2(0.62)
Alt.4				25(1.00)	1(0.62)
Alt.5					17(1.00)
**Interactions**	**Proposed**	**Alt.1**	**Alt.2**	**Alt.4**	**Alt.5**
Proposed	34(1.00)	10(0.68)	16(0.65)	11(0.63)	5(0.49)
Alt.1		30(1.00)	6(0.58)	17(0.84)	2(0.42)
Alt.2			62(1.00)	8(0.51)	10(0.47)
Alt.4				40(1.00)	4(0.45)
Alt.5					45(1.00)

Table B6: I-E interaction analysis: the OOI values of selected imaging features and interactions based on 100 random splittings using the proposed method.

Imaging features	OOI of main effects	OOI of interactions				
		Diagnosis age	Gender	AS	FG	MC
AreaShape_BoundingBoxMinimum_Y	0.71			0.51	0.47	
AreaShape_Extent	0.48	0.35				
AreaShape_Orientation	0.90		0.31	0.55	0.78	0.88
AreaShape_Zernike_8_6	0.80		0.36			
Count_identifyhemaprimarynuclei	0.71	0.47				
Granularity_13_ImageAfterMath	0.44		0.43	0.44		
Granularity_8_ImageAfterMath	0.58					
Granularity_11_ImageAfterMath.1	0.51	0.33	0.41	0.38		
Granularity_3_ImageAfterMath.1	0.82	0.82			0.35	
Granularity_5_ImageAfterMath.1	0.57	0.55	0.49		0.79	
Granularity_8_ImageAfterMath.1	0.68		0.44			
Granularity_9_ImageAfterMath.1	0.97	0.96		0.95		0.75
Texture_Correlation_maskosingray_3_01_256	0.70	0.55		0.44		
Texture_SumEntropy_maskosingray_3_01_256	0.39					
Texture_SumVariance_maskosingray_3_01_256	0.40					
Texture_Entropy_ImageAfterMath_3_01_256	0.49					
Texture_SumEntropy_ImageAfterMath_3_00_256	0.97	0.67				
Threshold_SumOfEntropies_identifyhemaprimarynuclei	0.33					
Threshold_WeightedVariance_Identifyeosinprimarycytoplasm	0.38					
Threshold_WeightedVariance_identifytissueregion	0.93	0.81	0.56		0.55	
Location_Center_X.2	0.87	0.46				
Neighbors_AngleBetweenNeighbors_Adjacent	0.36					
